# Structural dynamics reveal isolate-specific differences at neutralization epitopes on HIV Env

**DOI:** 10.1016/j.isci.2022.104449

**Published:** 2022-05-23

**Authors:** Edgar A. Hodge, Gajendra S. Naika, Sally M. Kephart, Adam Nguyen, Richard Zhu, Mark A. Benhaim, Wenjin Guo, John P. Moore, Shiu-Lok Hu, Rogier W. Sanders, Kelly K. Lee

**Affiliations:** 1 Department of Medicinal Chemistry, University of Washington, Seattle, WA 98195, USA; 2 Department of Pharmaceutics, University of Washington, Seattle, WA 98195, USA; 3 Division of Microbiology and Immunology, Weill Medical College of Cornell University, New York, NY 10021, USA; 4 Department of Medical Microbiology, Amsterdam UMC, University of Amsterdam, 1105 AZ Amsterdam, the Netherlands; 5 Biological Physics, Structure and Design Graduate Program, University of Washington, Seattle, WA 98195, USA

**Keywords:** Immunology, Microbiology, Structural biology

## Abstract

The envelope glycoprotein (Env) is the sole target for neutralizing antibodies against HIV and the most rapidly evolving, variable part of the virus. High-resolution structures of Env trimers captured in the pre-fusion, closed conformation have revealed a high degree of structural similarity across diverse isolates. Biophysical data, however, indicate that Env is highly dynamic, and the level of dynamics and conformational sampling is believed to vary dramatically between HIV isolates. Dynamic differences likely influence neutralization sensitivity, receptor activation, and overall trimer stability. Here, using hydrogen/deuterium-exchange mass spectrometry (HDX-MS), we have mapped local dynamics across native-like Env SOSIP trimers from diverse isolates. We show that significant differences in epitope order are observed across most sites targeted by broadly neutralizing antibodies. We also observe isolate-dependent conformational switching that occurs over a broad range of timescales. Lastly, we report that hyper-stabilizing mutations that dampen dynamics in some isolates have little effect on others.

## Highlights

• Structural dynamics are an important determinant of viral antigenic phenotype
• H/D-exchange mass spectrometry reveals isolate-specific variation in HIV Env trimers
• HIV Env trimer exhibits local conformational switching on widely varying time scales
• Stabilizing mutations that quench Env dynamics have highly isolate-dependent effects

## Graphical abstract

**Figure:**
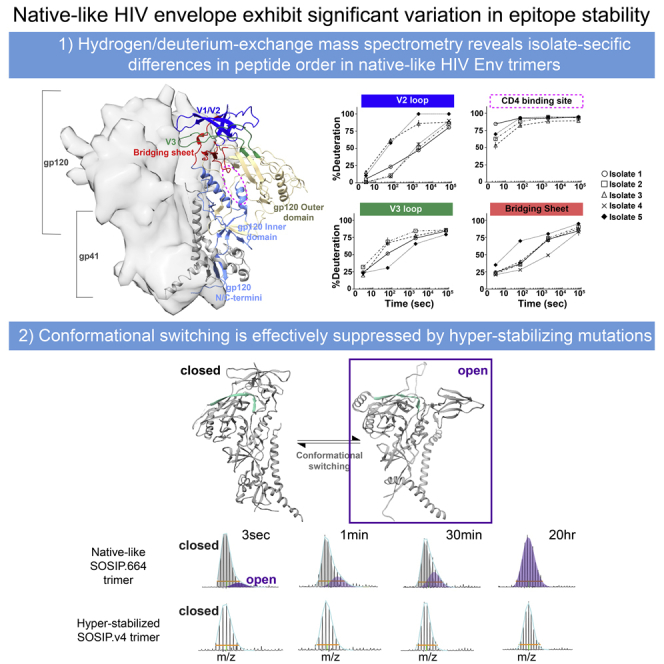

## Introduction

The HIV-1 envelope glycoprotein (Env) is a major determinant of viral tropism, pathogenesis, and neutralization. It is the only virally encoded protein displayed on the surface of the virus and the sole target for neutralizing antibodies (nAbs). Owing to intense immune pressures in each infected individual, Env evolves rapidly, making it the most variable part of the virus. Whereas high-resolution structures have provided a blueprint of Env’s protein and glycan architecture, the extent of Env structural and functional variation is only beginning to be understood (Julien et al., 2013; Li et al., 2020; Mangala Prasad et al., 2022; Pancera et al., 2014; Struwe et al., 2018). One fundamental but poorly characterized feature of Env that impacts its immune recognition is the high level of intrinsic dynamics embodied in the trimer at equilibrium even before CD4 receptor activation (Munro et al., 2014). Env exhibits dynamic conformational switching that exposes non-protective immunodominant epitopes and disrupts the presentation of conformational epitopes, whereas local structural flexibility can lead to disordered epitopes that reduce antibody affinities (Davenport et al., 2013; Kwong et al., 2002; Liang et al., 2016b). At present, we lack an understanding of the extent of differences in these cryptic traits among diverse HIV isolates owing to the challenges associated with measuring local dynamics in such a complex antigenic target.

An indication that different HIV isolates can exhibit significant differences in conformational dynamics was provided by single-molecule Förster resonance energy transfer (sm-FRET) experiments (Munro et al., 2014). Env trimers were shown to spontaneously interconvert between at least three distinct states, and the relative population bias differed significantly between a lab-adapted and a primary isolate. Dipole electron-electron resonance (DEER) spectroscopy analysis of Env trimers also revealed marked differences in the conformational states and structural heterogeneity displayed by engineered native-like “SOSIP” trimers from two isolates (Stadtmueller et al., 2018). Both sm-FRET and DEER spectroscopy, however, only provide information about the relative positioning of the respective fluorescent or spectroscopic probes. In order to draw a link between structure and antigenicity, it is necessary to probe local structure at specific epitope positions in different Env trimers.

Hydrogen/deuterium-exchange mass spectrometry (HDX-MS) offers a powerful means of probing local structural dynamics in proteins under native solution conditions (Hodge et al., 2020). This technique measures the kinetics of deuterium uptake from solvent by amide groups on the protein backbone. Amides that are occluded from solvent or protected by hydrogen bonding, such as in forming secondary structure, become deuterated more slowly, whereas those in dynamic regions of the protein exhibit more rapid deuteration. Coupled with proteolysis and mass spectrometry, one can monitor local protein dynamics with sequence-specific resolution. Our previous HDX-MS analysis of HIV Env demonstrated that isolate-specific differences in local epitope flexibility are apparent in isolated gp120 receptor binding subunits and in two early generation subtype A SOSIP trimers (Davenport et al., 2013; Guttman et al., 2015; Liang et al., 2016a; Verkerke et al., 2016). The studies of the gp120 subunits also demonstrated that local dynamics impact the binding of antibodies targeting conformational epitopes (Liang et al., 2016a).

Indeed, most HIV-1 antibody epitopes on native Env are conformational and in some cases involve quaternary structural features. Broadly neutralizing antibodies (bnAbs) with exceptional breadth are known to target conserved regions on Env such as the V1/V2 loop apex, base of the V3 loop, CD4 receptor binding site, fusion peptide, and the gp120/gp41 interface (Chuang et al., 2018; Wibmer et al., 2015). The majority of these bnAbs recognize conformational epitopes displayed by the closed, pre-fusion form of the trimer (Chuang et al., 2017; Guttman et al., 2015; Ward and Wilson, 2017).

**Figure 1:**
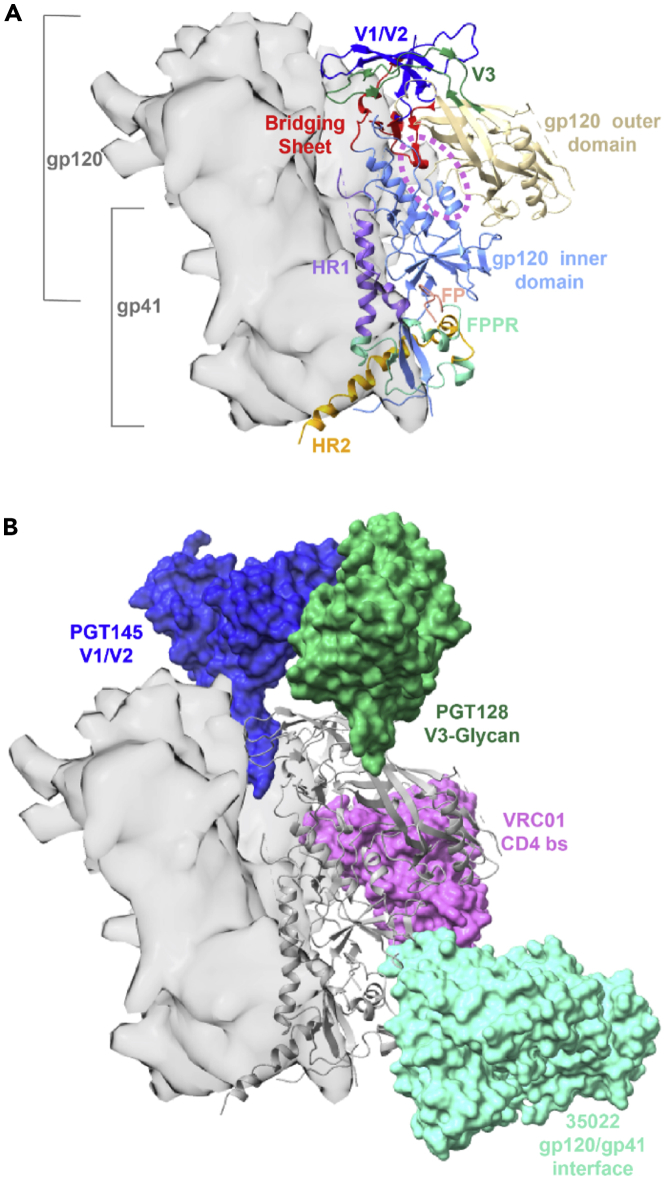
HIV Env domain organization and sites targeted by broadly neutralizing antibodies (A) Localized regions are highlighted on the Env BG505 EM structure (PBD 5ACO; one Env protomer is displayed in ribbon representation, whereas the other two protomers are displayed as a grey surface). Each of the three protomers consists of three gp120 receptor binding subunits at the apex, and three gp41 subunits at the base that house the fusion peptide. The gp120 outer domain is colored wheat, the gp120 inner domain is colored light blue, the CD4 binding site is outlined by a pink circle, the bridging sheet is colored in red, the V1/V2 apex is colored dark blue, and the V3 loop in green. In the base gp41 subunit, heptad repeat 1 (HR1) is colored purple, heptad repeat 2 (HR2) is colored orange, the fusion peptide (FP) is colored salmon, and the fusion peptide proximal region (FPPR) is colored teal. (B) Fabs from bnAbs that recognize four sites of vulnerability on Env are shown (PBD 5ACO for the Env ectodomain aligned with 5V8L, 5W6D, and 6VI0 PDB models in surface representation). bnAb PGT128 (green) targets the V3 base and N332 glycan, PGT145 (blue) binds the V1/V2 apex, VRC01 (pink) targets the CD4 binding site, and 35O22 (teal) binds the gp120/gp41 interface.

Here we utilized HDX-MS to characterize dynamic phenotypes in native-like SOSIP.664 Env trimers across five divergent strains to understand the impact of large sequence variations on structural dynamics and to determine whether differences in Env dynamics impact its recognition by antibodies. We examined trimers from the isolates BG505, B41, AMC008, JR-FL, and CE1176, all stabilized by the “SOSIP.664” modifications that help the trimer maintain a native-like pre-fusion form (Julien et al., 2013). These types of soluble trimers have been shown to closely mimic the native structure and antigenic profile of Env on virus (Julien et al., 2013; Ringe et al., 2019; Sanders et al., 2013a). Whereas cryo-EM structures show a high degree of structural homology (Lai et al., 2019; Ward and Wilson, 2017), HDX-MS analysis presented here reveals substantial dynamic differences that impact the structural ordering of nearly all bnAb epitopes present on the ectodomain (Figure 1) including the V1/V2 apex targeted by bnAbs such as PGT145 and VRC26 (Doria-Rose et al., 2014; Lee et al., 2017a); the CD4 binding site targeted by VRC01 class bnAbs (Wu et al., 2011); the V3 loop targeted by PGT121 class bnAbs (Mouquet et al., 2012; Pejchal et al., 2011); and the gp120/gp41 interface that includes the fusion peptide and is the target of bnAbs such as 35O22, PGT151, and VRC34 (Huang et al., 2014; Kong et al., 2016; Xu et al., 2018).

We also observed conformational sampling between deuterium-exchange protected and exposed states at multiple sites and found that the sampling timescales were not correlated across the trimer, indicating that regional trends in the sub-domain behavior predominate rather than all parts of the trimer switching in concert. This aspect of Env’s architecture may impact mechanisms of neutralization and cooperativity of antibodies that target distinct epitopes across this complex antigenic target.

Lastly, a recent trend in viral immunogen design has been toward increasing stabilization of the closed conformational state, reducing conformational flexibility and increasing antigenicity (de Taeye et al., 2015; Hsieh et al., 2020b; McLellan et al., 2013; Pallesen et al., 2017; Torrents de la Pena and Sanders, 2018). Since the original SOSIP modifications were introduced (Binley et al., 2000; Sanders et al., 2002, 2013b), additional stabilizing mutations have been tested with the aim of increasing the trimer stability and reducing the exposure to non-protective immunodominant epitopes such as the V3 loop and portions of the V1/V2 loops (de la Peña et al., 2017; de Taeye et al., 2015; Rawi et al., 2020). We thus also examined the consequences for antibody binding kinetics and affinity, resulting from suppressing protein dynamics by introducing additional hyper-stabilizing mutations into the Env trimer.

Collectively, we find that isolate-specific differences in Env structural dynamics impact the display and recognition of conserved epitopes by bnAbs. With this type of information, we can optimize the design of stabilized immunogens to suppress dynamics as well as aid in the selection of immunogens with desirable structural and dynamic properties.

## Results

### Isolate-specific differences in trimer structural dynamics

In order to investigate isolate-specific differences in Env conformation and local dynamics, HDX-MS was used to track the dynamic behavior of peptides in SOSIP.664 trimers from five HIV-1 isolates: subtype A BG505, subtype B AMC008, B41, and JR-FL, and subtype C CE1176 (Daar et al., 1990; deCamp et al., 2014; Hoffenberg et al., 2013; Pugach et al., 2015). With the exception of AMC008, these isolates have been characterized as “tier 2” resistant to neutralization by pooled sera from HIV-infected individuals (deCamp et al., 2014; Seaman et al., 2010). AMC008, by contrast, is a tier 1B, moderately neutralization-sensitive isolate (de Taeye et al., 2015). Pairwise sequence variation across isolates ranged from 12 to 27%.

**Figure 2:**
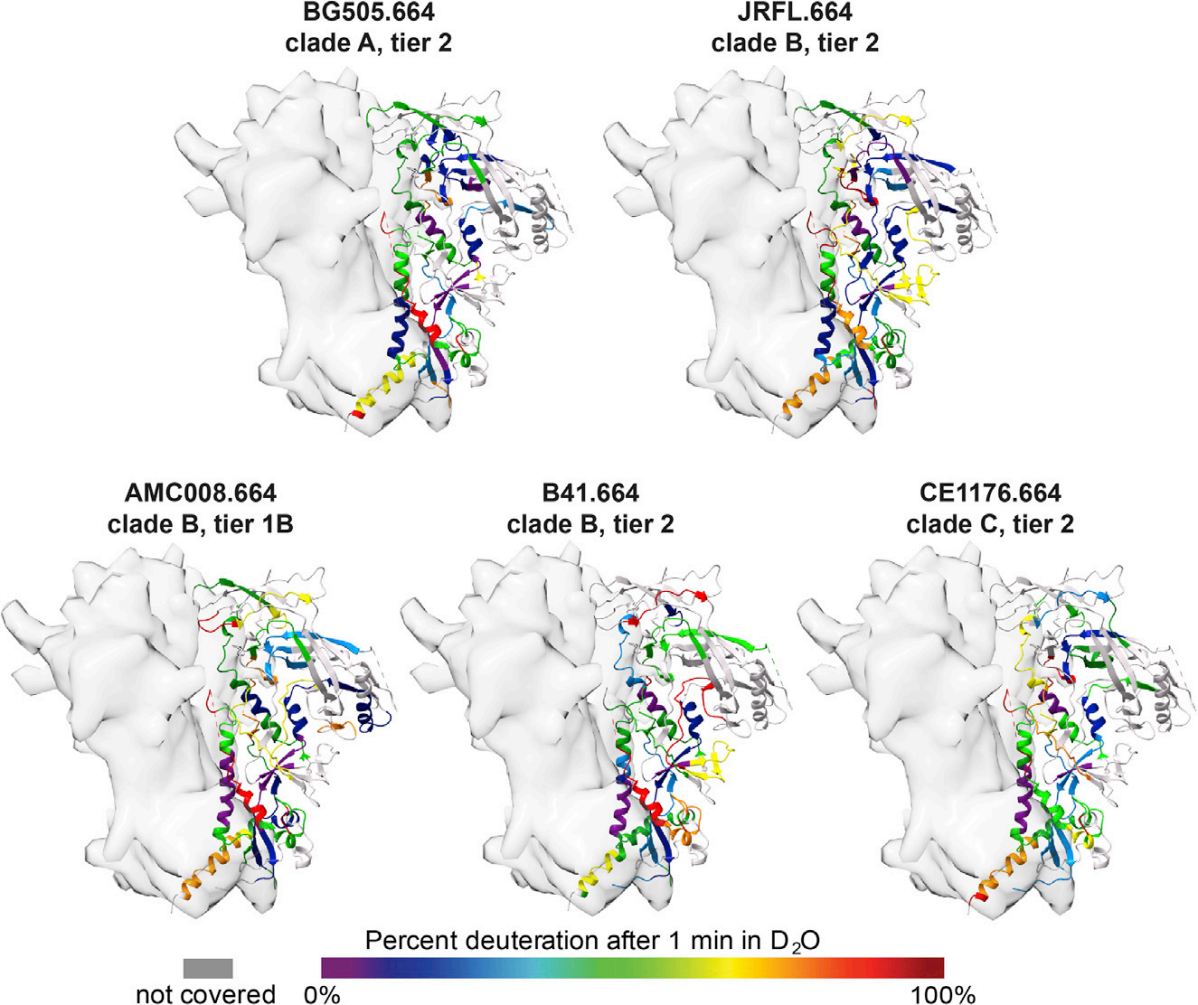
HDX heatmaps exhibit isolate-specific differences in Env structural dynamics in regions including epitopes targeted by broadly neutralizing antibodies, related to Data S1 Percent deuteration after 1 min of exchange for the five Env in our panel mapped on to the 5ACO Env structure (2/3 protomers shown in gray surface representation, one protomer displayed as a ribbon color coded according to the HDX heatmap). Cooler colors indicate regions that are more protected from exchange, and warmer colors indicate regions that are more exposed and take up deuterium more readily.

HDX-MS heatmaps for each isolate in Figure 2 show the deuterium exchange levels per peptide after 1-min incubation in deuterated buffer, providing an overall dynamic portrait of each Env trimer ectodomain; complete reporting of all HDX-MS data can be found in Figures S6–S8, Table S1, and Data S1. Figure S1 describes the HDX analysis workflow. Overall coverage of each trimer was roughly 60%, with the largest gaps in coverage resulting from reduced intensity of glycopeptides in the variable loops (Stavenhagen et al., 2013). We note that the heatmaps include non-homologous peptides; thus, these overview data are best interpreted qualitatively in terms of regional differences in dynamics. Many regions across each structure showed similar backbone amide protection that suggests the conservation of structural ordering; however, key regions with substantial differences in dynamics were also observed covering the base of V1, V2, and V3 loops, and the HR2 motif at the trimer’s base (Figure 2). Many of these regions showing differences span sites of vulnerability targeted by bnAbs (Figure 1).

**Figure 3:**
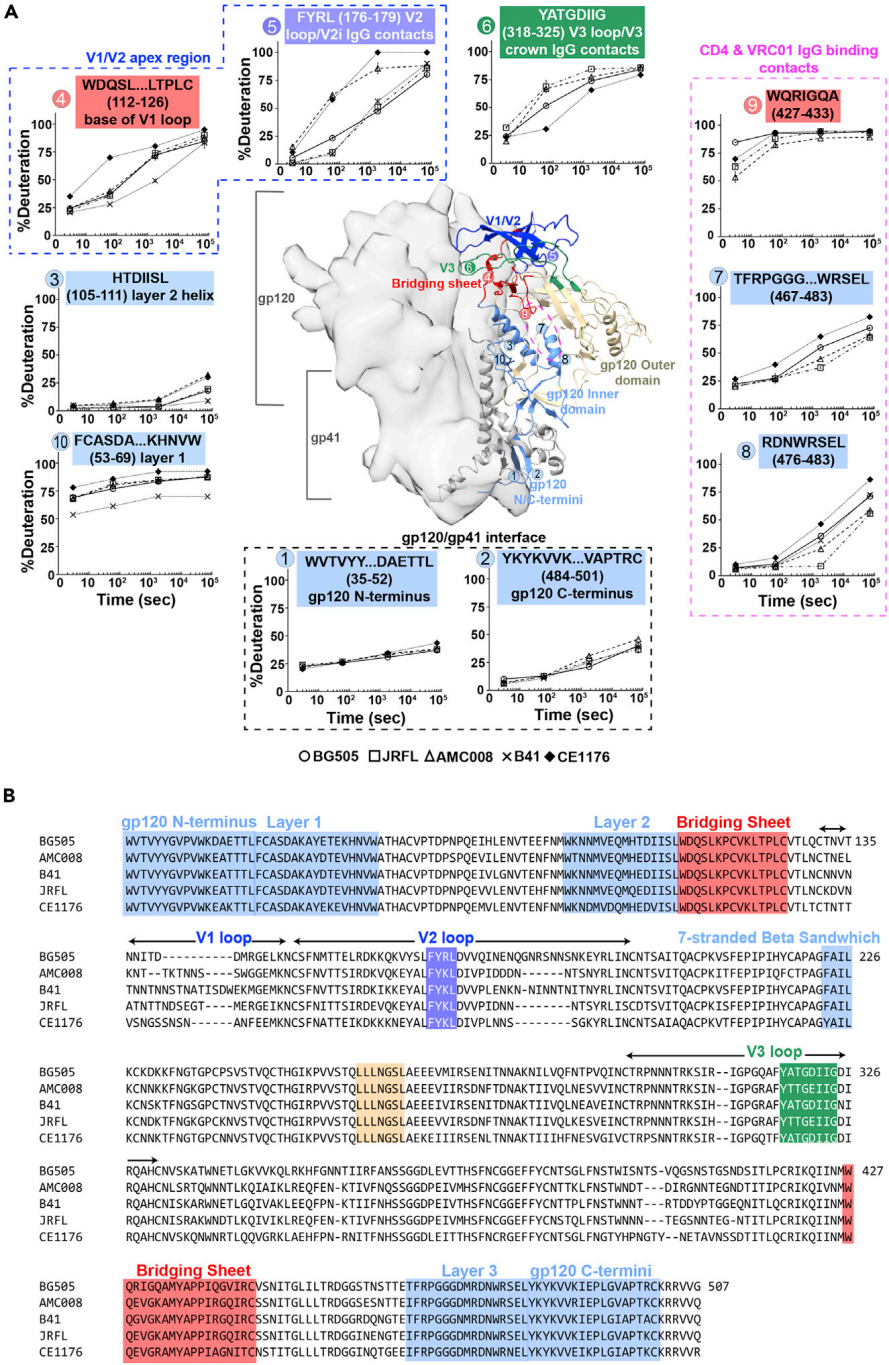
Dynamic differences in gp120 subunits within SOSIP.664 trimers mapped by HDX-MS (A) Deuterium uptake plots of homologous peptides in gp120 are shown. Each HDX uptake plot consists of the average percent deuteration of at least two replicates after 3 s, 1 min, 30 min, and 20 h of exchange normalized to a fully deuterated control with error bars displaying standard deviation. All homologous peptides contain the same number of exchangeable backbone amides across the five strains (BG505 circle, JR-FL square, AMC008 triangle, CE1176 diamond, and B41 indicated by x). In each uptake plot, the homologous peptide sequence (from reference BG505 isolate) is shown and color-coded to match the region on the SOSIP structure and sequence (B). All gp120 inner domain peptides (including layers 1–3) are colored light blue, V1/V2 loops dark blue, V3 loop green, bridging sheet red, the outer domain wheat, and the CD4 binding site is outlined by a pink circle.

**Figure 4:**
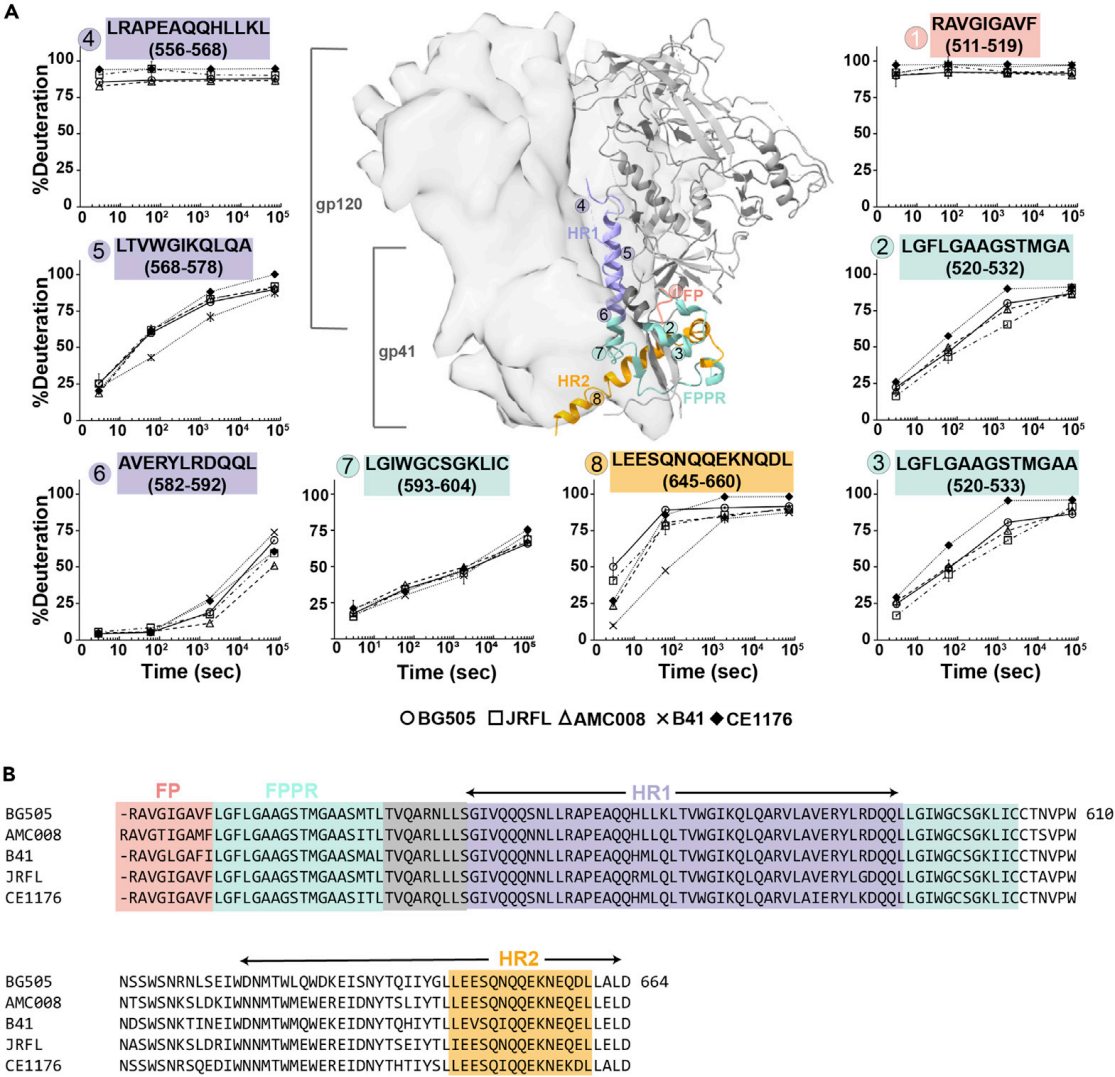
Dynamic differences in gp41 subunits within SOSIP.664 trimers mapped by HDX-MS (A) Deuterium uptake plots of homologous peptides in gp41 are shown. Each HDX uptake plot consists of the average percent deuteration of at least two replicates after 3 s, 1 min, 30 min, and 20 h of exchange normalized to a fully deuterated control with error bars displaying standard deviation. All homologous peptides contain the same amount of exchangeable backbone amides across the five strains (BG505 circle, JR-FL square, AMC008 triangle, CE1176 diamond, and B41 indicated by x). In each uptake plot, the homologous peptide sequence (from reference BG505 isolate) is shown and color-coded to match the region on the SOSIP structure and sequence. (B). Homologous peptides found spanning the fusion peptide are colored salmon, the fusion peptide proximal region (FPPR) in cyan, HR1 is colored purple, and HR2 is colored orange.

In cases where homologous peptides could be tracked in the different trimers, quantitative comparisons of deuterium uptake kinetics among isolates were possible (Figures 3 and 4). Trimers from the well-characterized isolate, BG505 (de Taeye et al., 2015; Guttman et al., 2014), were purified and exchanged as a reference control alongside each batch of the non-BG505 trimer. We set a threshold that any apparent difference in deuterium uptake between isolates exchanged independently was considered significant if the differences were greater than the variability observed in that homologous peptide between BG505 biological replicates from independent protein preparations (Figure S2); we note the experimental error from technical replicates (plotted as error bars in deuterium uptake plots) is far smaller than this threshold.

Exchange reactions were carried out immediately after protein purification without subjecting the fragile trimer specimens to freeze-thaw cycles. To verify the homogeneity of the trimers and ensure that the samples were free of dissociated or misfolded species, samples were analyzed by blue native-PAGE, SDS-PAGE, DLS, and negative stain EM (Figure S3).

We further confirmed that the samples were composed of native-like pre-fusion trimers by examining HDX signatures for peptides covering the gp120 N and C-termini (Figure 3A, peptides 1 and 2) that showed significant protection from deuterium exchange, consistent with the trimers embodying well-formed gp120-gp41 interfaces (Guttman and Lee, 2013; Guttman et al., 2014; Verkerke et al., 2016). Other well-ordered regions of the trimers also showed similar exchange protection. For example, the inner domain layer 2 of gp120 was relatively protected in all isolates over time points up to 20 h (peptide 3, Figure 3A), consistent with the peptide adopting a well-ordered helix as observed in high-resolution structures. Differences in protection were detected at 20 h, however, where transient disruption of backbone amide H-bonds led to greater deuteration over time in some isolates compared with others. Here this inner domain segment was more protected in the isolates JR-FL and B41, and slightly less protected in CE1176 and the tier 1B isolate AMC008.

Other regions across the gp120 subunit showed larger differences in local peptide dynamics (Figure 3), for instance, the V1/V2 apex region including peptides 4–5. This region is a target of bnAbs, such as PGT145, PG9, and VRC26 (Doria-Rose et al., 2014; Lee et al., 2017b; Sok et al., 2014). The base of V1 (peptide 4), which also forms part of the bridging sheet upon CD4-induced structural rearrangements, was considerably more protected in B41 but highly dynamic in CE1176. At the trimer apex, the V2 loop likewise was highly dynamic in CE1176 as well as AMC008 as reflected by peptide 5 with the sequence FYRL that lies directly upstream of a proposed integrin-binding site and the V2i antibody epitope (V2 epitope overlapping the integrin-binding site). Interestingly, all FYR/KL peptides start off similarly protected, but their backbone amide protection varies dramatically with time, suggesting a similar ground-state level of amide protection. The differences in the rate of deuterium uptake for this peptide among different Env trimers would indicate differences in the transient backbone exposure of the V2 loop, departing from the more protected ground state configuration. Indeed, the V2 loop has been shown to be capable of adopting different conformations (a four-stranded beta barrel, a partial helical-loop conformation, and a more protected beta sheet conformation in the open state) (Powell et al., 2017). Envs with greater sampling among such conformations would take on deuterium more readily and the structural fluctuations would likely influence how V2i antibodies and integrins bind this region.

The third variable loop (V3), one of the most immunodominant regions on Env, is sequestered underneath V2 in all available closed, pre-fusion trimer structures. It has been suggested, however, that V3 can “flicker” out from underneath V2 without disrupting the V1/V2 apex ordering (Pan et al., 2015; Powell et al., 2017). V3 loop dynamics control the accessibility of the V3 crown/tip epitope to antibodies that bind this region. From HDX-MS analysis, significant differences in V3 exchange kinetics were observed. CE1176 exhibited the most protected V3 loop whereas JR-FL exhibited a rapidly exchanging V3 loop (peptide 6, Figure 3). AMC008, while paralleling CE1176’s dynamic profile in many areas, broke with CE1176 in exhibiting a V3 loop that more frequently samples an exchange-accessible conformation.

Interestingly, in similar fashion to the FYR/KL peptide in V2, despite differences in overall deuterium exchange kinetics for these peptides in V1, V2, and V3, they all exhibited similar levels of exchange at the earliest time point but then diverged over the time courses. These trends indicate that these loops adopt similar ground-state configurations in the trimers from the panel of isolates, even in the relatively dynamic CE1176 and AMC008 trimers, but they exhibit significant differences in their frequency of sampling more exposed conformations, giving rise to differences in the slope of the deuterium uptake plots (Hodge et al., 2020).

The CD4 binding site, including peptides 7–9, also exhibited significant differences in structural dynamics. Greater protection was observed in AMC008 and JR-FL relative to BG505 and B41 for two homologous peptides in gp120 containing residues that are direct binding contacts with CD4 and the bnAb VRC01 (peptides 7 and 8; Figure 3). Conversely, CE1176 is more dynamic in these same regions. The trend with a similar exchange at early time points and divergent exchange kinetics over time mirrors what was observed for the variable loops in the apex, again suggesting a similar ground-state peptide environment with isolate-specific differences in the frequency of sampling more exposed configurations.

By contrast, peptide 9, which forms a beta-hairpin turn that projects toward CD4 when it is bound, at early time points showed divergent deuteration levels with BG505 taking on nearly saturating levels of deuteration and AMC008, exhibiting more protection (Figure 3). The variance in deuterium exchange at the earliest time points for this key CD4 binding site peptide, indicates that this site is intrinsically more disordered or accessible at equilibrium in BG505 than the other Env trimers.

Other peptides also show differences in deuterium exchange at the earliest time points, highlighting intrinsic differences in local ordering across isolates. This is observed, for example, in the pivotal gp120 inner domain layer 1 peptide 10 that has been shown to help regulate CD4 activation; this also is a region where stabilizing modifications can shift the conformational equilibrium of Env toward the closed state (Finzi et al., 2010; de Taeye et al., 2015).

Whereas the largest isolate-specific differences in local structural dynamics were concentrated in the gp120 subunit, peptides in gp41 also exhibited structural differences in dynamics despite the relatively high sequence conservation of this subunit. The *N*-terminal fusion peptide of gp41 (peptide 1, Figure 4) was rapidly deuterated in all strains, in agreement with this region’s high degree of flexibility and solvent accessibility suggested by a lack of density in nearly all structures of unliganded Env (Ananthaswamy et al., 2019; Kumar et al., 2019; Mangala Prasad et al., 2022). By contrast, the adjacent fusion peptide proximal region (FPPR, peptides 2 and 3; Figure 4) showed gradual deuterium uptake over 20 h, which suggests that it samples exposed conformations infrequently, and the extent of the dynamic sampling differed among isolates. Peptides at the *N*-terminal end of HR1 (e.g. peptide 4, Figure 4) were highly dynamic in all strains. This region is disordered in most SOSIP crystal and cryo-EM structures likely owing to the helix disrupting proline point mutation (I559P) designed to prevent transition to a rigid helix in the post-fusion form. The segment was observed to exist as a rigid helix in a non-SOSIP pre-fusion JR-FL strain Env structure (Lee et al., 2016; Pan et al., 2019). Notably, whereas high-resolution crystal and cryo-EM structures indicate that the core HR1 segment is part of a stable helical bundle, our HDX-MS data suggest that the degree of structural order of this helix can vary across isolates. For example, in the B41 isolate, the central stretch of HR1 (peptide 5, Figure 4) shows greater exchange protection than the other four, which appear similarly behaved. The *C*-terminal end of the HR1 helix (peptide 6) is similarly protected from exchange in all strains at the earliest two time points, but isolate-specific differences became apparent at later time points. At the very base of the trimer, the dynamic profile of HR2 suggests differences in helix stability across the isolates, where, again, B41 exhibited the most exchange-protected HR2 segment (peptide 8).

From the comparison of homologous peptides, it is clear that divergent Env trimers exhibit a broad range of local structural dynamics at key functional and antigenic sites despite appearing nearly identical in static structures.

### Isolate-specific differences in conformational switching

We also investigated whether HDX-MS could identify signatures of large-scale conformational switching across Env that may relate to the dynamic changes reported by sm-FRET (Hodge et al., 2020; Munro et al., 2014). Signatures of conformational transitions manifest as broadened or bimodal mass spectral envelopes, indicative of the peptide populating states with differences in backbone amide protection. Bimodal spectra were fit to two populations using the HX-Express analysis program (Guttman et al., 2013; Weis et al., 2006). Bimodal spectra arise from slow (relative to the deuterium labeling rate) concerted motions where multiple backbone amides become deuterated in an exchange-competent state before the peptide has a chance to return to the more protected state. In this exchange regime (so-called EX1 kinetics), the rate of exchange equals the rate of conformational opening to a state with greater amide accessibility to solvent (Benhaim et al., 2019; Nielsen et al., 2019).

**Figure 5:**
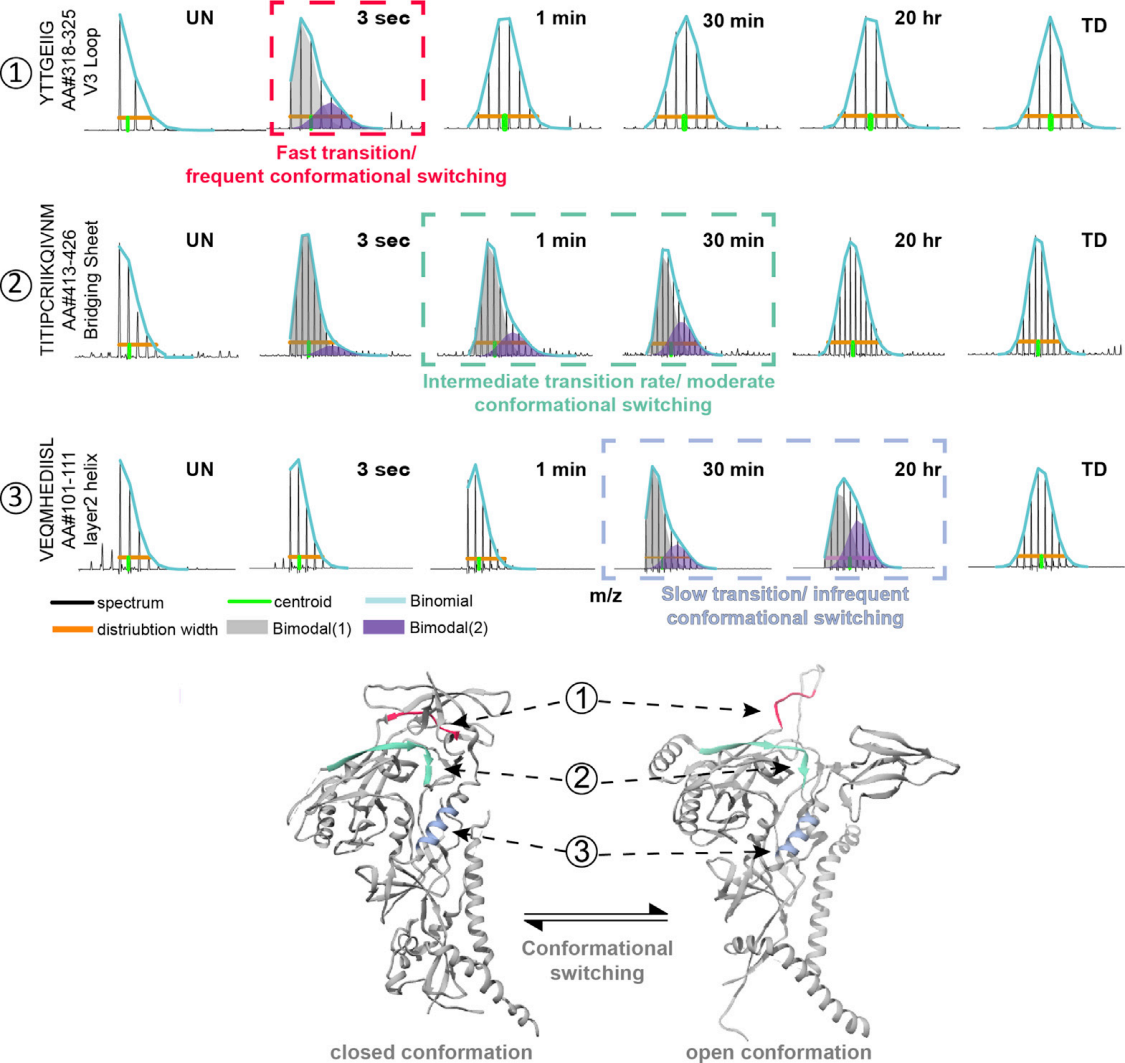
Bimodal mass spectra in three AMC008 gp120 peptides show spontaneous local conformational switching that occurs on different timescales Conformational sampling rates occur frequently (red), over intermediate timescales (green), and infrequently (blue) for peptides in different regions. Bimodal mass envelopes were binomially fit to two populations; a more protected, lighter in mass population in grey, and a more exposed, deuterated population in purple. The three peptides (a V3 peptide, a peptide in the gp120 inner domain helix 2, and a peptide that is part of the bridging sheet/ CD4i epitope) are highlighted according to the relative rate of opening on the structure of Env in the closed conformation (PDB 5ACO with one protomer in ribbon representation) and on a theoretical structure of Env in an open conformation (PDB 3J70 with one protomer in ribbon representation) to illustrate a hypothetical example of how conformational switching may alter localized structure and give rise to bimodal spectra.

In the Env HDX-MS data, if bimodal spectra were detected for a given peptide and the overall population transitioned to the exposed state within 1 min, those were categorized as fast transitions driven by frequent conformational switching. If the protected population exhibits signs of a mixed population even after hours (as evidenced by persistent bimodal spectra at the late time points), those transitions were categorized as slow or infrequent switching events. A number of peptides also exhibited intermediate timescales for the kinetics of population transitions. Examples of peptides that show bimodal spectra indicative of conformational sampling on the different timescales are presented in Figure 5.

**Figure 6:**
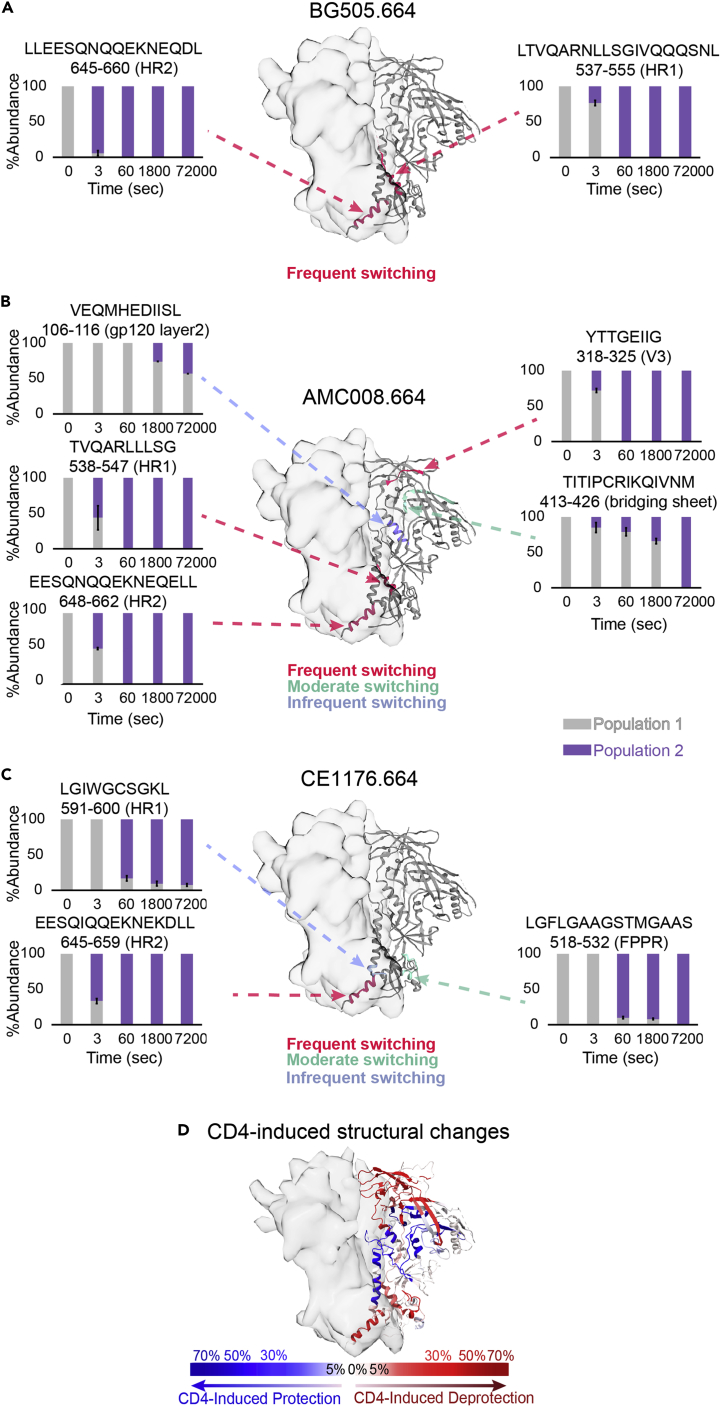
Localized regions across the trimer base and apex undergo spontaneous conformational transitions over a broad range of timescales and in an isolate-specific fashion Purple and grey bars indicate average population percentages of conformational states for specific peptides indicated in BG505 (A), AMC008 (B), and CE1176 (C) SOSIP.664 trimers with standard deviation error bars (also see Figure S4). As in Figure 5, peptides sample exposed conformations on a range of time scales ranging from very frequent (red; bimodals only observed in 3-s time points) to very infrequent (blue; bimodals persist after hours). (D) Most peptides exhibiting bimodal behavior align with regions involved in CD4-induced conformational changes as shown in HDX-MS heatmap showing the percent difference in the exchange of unbound and CD4-bound BG505 SOSIP characterized by Guttman et al. (2014).

In AMC008, bimodal spectra were detected for the V3 peptide (Figures 5 and 6B, Figure S4), but only at the earliest time point, after which the populations converged to a more exposed state. This suggests V3 in AMC008 undergoes a rapid sampling between protected and exposed states. Bimodal spectra from a peptide spanning residues 413–426 in the bridging sheet report a transition to a more exposed state on an intermediate timescale. By contrast, the helix in layer 2 of AMC008 gradually samples a more exposed state over a long timescale, where even after 20 h a population that has not sampled an exposed state can be resolved. Overall, AMC008 displayed the greatest number of bimodal spectra indicative of conformational transitions across the apex and trimer base, whereas B41 and JR-FL appear to be the most conformationally fixed with no bimodal peptides identified across the timescales we probed. Isolate-specific differences in structural and conformational dynamics thus extend beyond local peptide flexibility to include the movement of larger sub-domains that sample distinct conformational states over a broad range of timescales.

The majority of peptides exhibiting bimodal profiles were found in the gp41 subunit (Figure 6). Two distinct populations in the mass spectra for peptides located in gp41’s HR2 were resolvable only at the earliest time point for BG505, CE1176, and AMC008 trimers (Figures 6A–6C and S4). Similarly, peptides at the *N*-terminal end of HR1 exhibited bimodal spectra in early time points in BG505 and AMC008 (Figures 6A and 6B) suggesting this region may frequently interconvert between a well-ordered helix and a more backbone accessible conformation. CE1176 displayed a broader range of timescales of structural transitions in gp41 compared with BG505 and AMC008. The FPPR of CE1176 transitions from a protected state to a more exchange-competent state on an intermediate timescale, up to 30 min (Figure 6C). The more *C*-terminal end of HR1 in CE1176 by contrast appears to sample two distinct conformations considerably less frequently than the FPPR, where even after 20 h a fraction of the protected population did not yet transition to the exposed state (Figure 6C). Based upon the wide range of timescales observed for conformational switching at different sites in the trimer, the individual regions do not necessarily appear to be moving in concert with each other. Instead, more modular, regional conformational sampling is occurring.

Despite the lack of apparent coordination between sites that exhibit conformational switching, the localization of these peptides is consistent with regions that respond to CD4-induced changes that we previously mapped out by HDX-MS (Guttman et al., 2014). For example, in response to CD4 binding, the FPPR and HR2 became less protected, whereas the *C*-terminal end of HR1 became more protected in the transition from the closed to the CD4-induced open state (Figure 6D) (Guttman et al., 2014). The trends and clustering of bimodal HDX-MS spectra throughout gp41 are also in agreement with DEER spectroscopy work that reported conformational heterogeneity in gp41 (Wang et al., 2018).

### Stabilizing mutation effects on local dynamics and conformational switching

Given the differences in local epitope dynamics and order we had observed, we sought to investigate the effect of Env structural dynamics on antibody affinity. Env sequence differences between the divergent isolates confound direct quantitative comparisons, however. To overcome this limitation, we examined the structural dynamics and antigenic profiles of SOSIP.664 compared against isolate-matched hyperstabilized forms of three of the Env — AMC008, BG505, and B41 – that showed dramatic differences in intrinsic structural and conformational dynamics. CE1176 was also examined; however, the stabilizing mutations described below failed to produce sufficient native-like trimer for analysis. HDX-MS was first used to test the ability of additional stabilizing mutations to abrogate localized trimer dynamics in these three Envs. Next, biolayer interferometry (BLI) was used to characterize the antigenic effect of those changes in epitope flexibility.

We previously examined differences in dynamics between minimally engineered SOSIP.664 trimers and SOSIPv4.1 and 4.2 (mutations described below) for the subtype A BG505 isolate (de Taeye et al., 2015). Here we confirmed the stabilizing v4.1 and v4.2 mutation effects on dynamics first in BG505 as a control, and then extended the analysis to the highly dynamic Env AMC008 as well as the relatively well-ordered B41 trimer. Because AMC008 and B41 previously were shown to form well-folded trimers with good yields after inserting SOSIPv4 mutations, and they exhibited such starkly different dynamic phenotypes, we decided they would serve as informative cases to compare and contrast how abrogated epitope dynamics influence antibody recognition. The SOSIPv4 changes added on top of the SOSIP.664 modifications include E64K (v4.1) or H66R (v4.2) in the gp120 layer 1 inner domain to stabilize the closed state, A316W (both in v4.1 and v4.2), to lock the V3 loop under the trimer apex, and I535M and L543N in gp41 to match the residues in the base of BG505, which have been shown to boost trimer yield and thermostability (de Taeye et al., 2015).

In agreement with our previous analysis of BG505, we confirmed that the v4 mutations in the BG505 reference trimer resulted in only minor changes in dynamics proximal to the stabilizing mutations (Figure S6) (de la Peña et al., 2017; de Taeye et al., 2015). In the context of B41, which generally exhibits greater local order relative to BG505, the v4 modifications likewise yielded minimal differences in dynamics across the three SOSIP versions (Figure S7). Even a peptide containing the E64K/H66R mutation in the gp120 layer 1 inner domain did not exhibit a major change in protection as a result of the SOSIPv4 residue substitutions in B41. Although no significant changes in dynamics were observed in BG505 and B41, it has been shown the thermostability and yields slightly increase during v4 stabilization (de Taeye et al., 2015).

**Figure 7:**
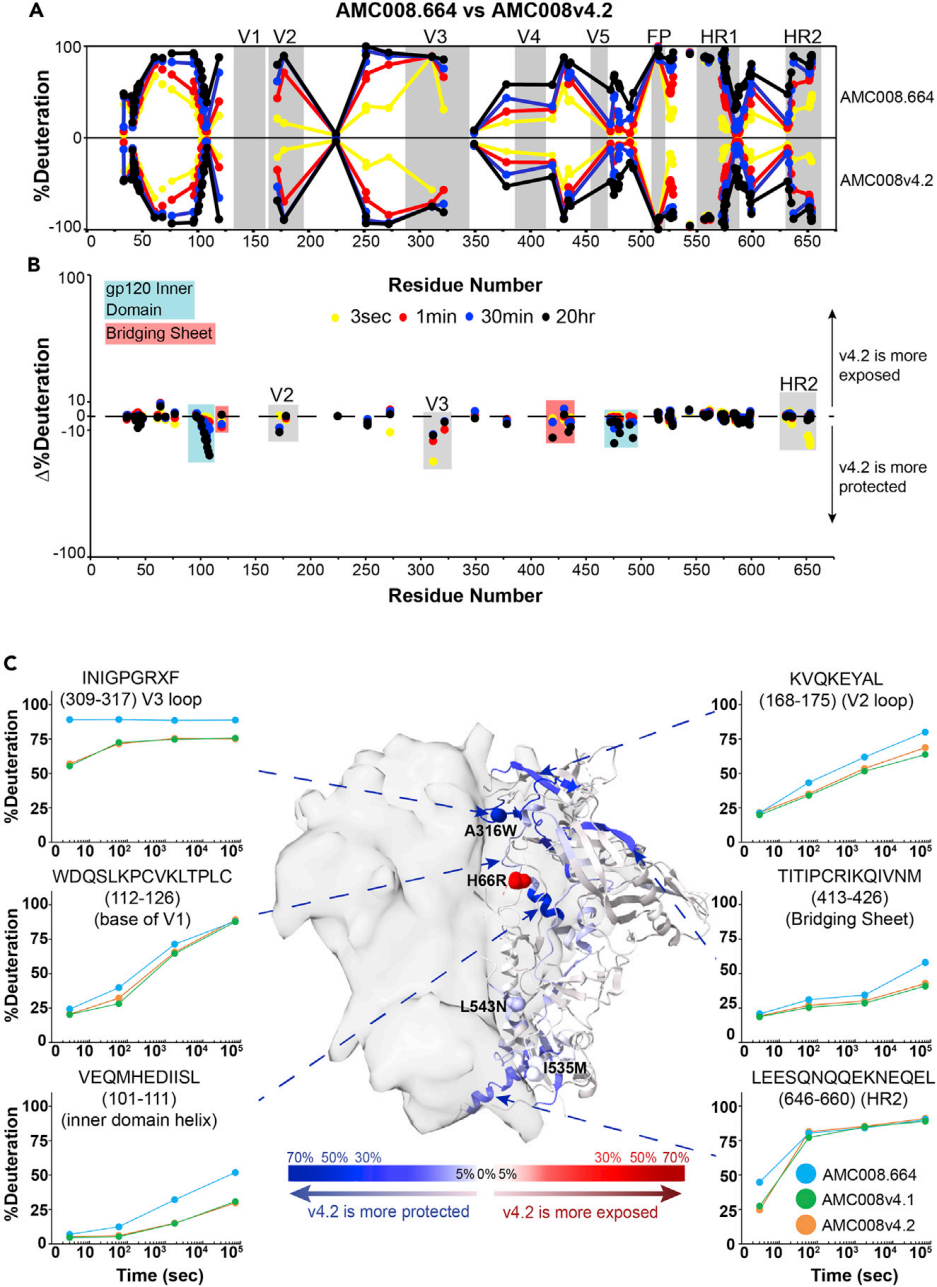
Stabilizing mutations quench dynamics across the AMC008 structure (A) HDX-MS butterfly plot showing the profile of dynamics for SOSIP.664 (top) and SOSIPv4.2 (bottom) trimers. The mid-point of each homologous peptide is plotted to show the deuterium uptake at each time point (3 s yellow, 1 min red, 30 min blue, and 20 h black). (B) Differential plot showing the percent difference in exchange between AMC008.664 and v4.2. Points below the axis are peptides that are more protected in the v4.2 construct, and peptides above the axis are more exposed in the v4.2 construct (also see Figure S8). (C) Sum of the differences in backbone amide dynamics between AMC008.664 and v4.2 across time are mapped onto the BG505 SOSIP structure (PDB 5ACO). Regions colored blue are more protected from the exchange in v4.2, and regions colored red are the exposed ones. Residues with space filling rendering indicate positions of v4.2 stabilizing mutations including H66R in the gp120 inner domain layer 1, A316W in the V3 loop, I535M, and L543N in gp41. Deuterium uptake plots comparing differences in backbone amide protection across time (3 s, 1 min, 30 min, 20 h) in AMC008.664, v4.1, and v4.2 (cyan, green, and orange circles, respectively). Each point on the plot is the average percent deuteration of two replicates normalized to a fully deuterated control with standard deviation bars. An X in the peptide sequence is used to indicate mutation sites.

By comparison, the AMC008 isolate showed a dramatic response to v4 stabilizing modifications (Figures 7 and S8). Increases in deuterium exchange protection were observed in the gp120 inner domain, the bridging sheet, the V2 loop, and the V3 loop, including a peptide spanning the A316W mutation site. The increased protection observed in the V3 loop peptides suggests the A316W mutation indeed helped sequester the V3 loop. Whereas AMC008 SOSIP.664 exhibited multiple regions with bimodal spectra, the majority of regions with coverage in v4.1 and v4.2 constructs exhibited unimodal spectra suggesting the v4 constructs were locked into one conformational state (Figure S5). The exception to this suppression of conformational sampling was at the *N*-terminal end of HR1 (residues 538–546), which contains the L543N mutation and exhibited a persistent bimodal spectrum at the earliest time point.

### Antigenic consequences of suppressed dynamics in Env trimers

In order to link antigenicity with the effects of reduced structural dynamics, a panel of antibodies specific for different conformational states and regions on Env were used for BLI experiments. In addition to antibodies (Abs) specific for the closed conformation (PGT145 and PG16), the open conformation (17b), and a partially open conformation (b12), three V3 tip binding Abs (447-52D, 3869, and 3074) and two V2i Abs (830A and 2158) were used here (Kwong et al., 1998; McLellan et al., 2011; Zhou et al., 2007). 447-52D and 3074 have been called “best in class” V3 Abs, and the three V3 tip Abs are broadly reactive and can neutralize tier 1 and 2 pseudo-typed HIV viruses across diverse subtypes (Han et al., 2019; Hioe et al., 2010; Pan et al., 2015). mAbs 830A and 2158 bind discontinuous epitopes that overlap a potential integrin binding site on the V2 loop and fall into the class of Abs that correlated with protection against HIV infection in the RV144 vaccine trial but are not potent neutralizers (Duerr and Gorny, 2019; Mayr et al., 2013; Pan et al., 2015).

**Figure 8:**
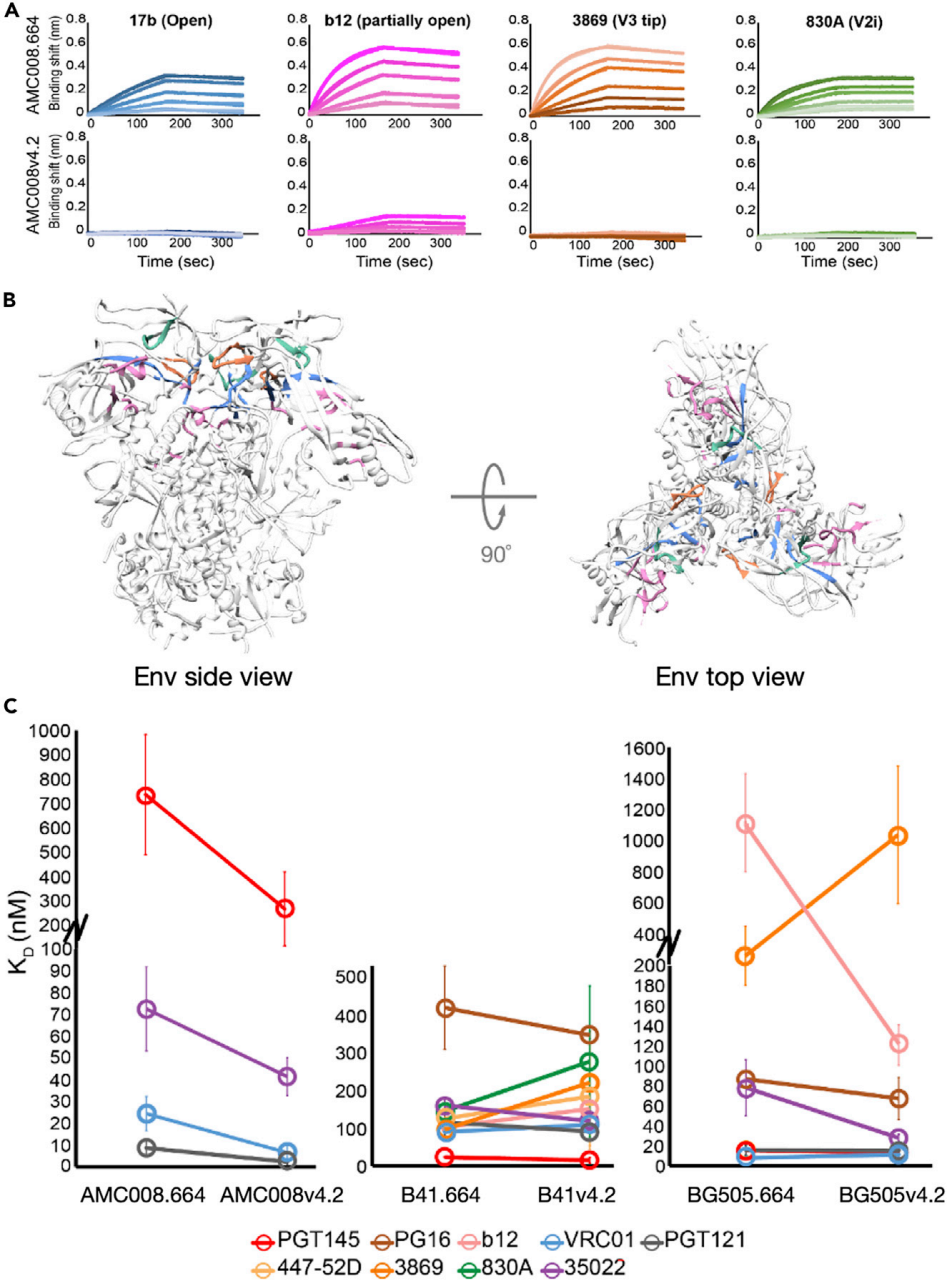
Antigenic consequences of quenched dynamics in three divergent Env trimers (A) BLI sensorgrams reporting on monoclonal antibody (mAb)-Env binding for mAbs that are specific for the open conformation (17b), a partially open conformation (b12), the tip of V3 (3869), and the V2i epitope (830A) of AMC008.664 and the v4.2 construct shows the antigenic effects of reduced flexibility (also see Tables S2–S4). The color of each sensorgram corresponds to the epitope of the antibody that is highlighted on the SOSIP structure in the closed conformation (PDB 5ACO) (B); side view (left) and top-down view (right). (C) For antibodies where K_D_s could be determined, line plots were used to compare differences in binding affinities between the minimally engineered SOSIP and the v4.2 construct across AMC008, B41, and BG505. Binding affinities are the average of two independent experiments and error bars are the standard deviations.

Across all the isolates, the antibody 17b against the CD4-induced coreceptor binding site was only able to bind the AMC008.664 construct, but its binding was abrogated in the v4.2 trimer, consistent with the modifications stabilizing a closed conformation of the trimer. Likewise, nAb b12 against a partially open form of the CD4 binding site was able to bind the AMC008.664 trimer relatively tightly, but the binding was suppressed in AMC008v4.2. All three V3 tip binding and V2i antibodies showed abrogated binding to the AMC008v4.2 construct that demonstrates that these epitopes were sequestered or less frequently exposed in the stabilized constructs, which is in agreement with the loss of localized regions exhibiting bimodal spectra in the HDX data (Figure 8A, Tables S2–S4).

In contrast to the dramatic shift in binding profiles for the antibodies above, the bnAbs VRC01, 35O22, and PGT121 demonstrated modest changes in binding between AMC008.664 and v4.2 constructs, with increases in K_D_ on the order of two- to four-fold, suggesting they are less susceptible to conformational variation in their antigenic targets. For the V1/V2 apex-targeting bnAb PGT145, though affinity increased against the stabilized v4.2 SOSIP, the antibody binding remained relatively weak. This is consistent with the still relatively dynamic, though slightly more protected, nature of the V2 apex in AMC008v4.2 trimers.

In the highly stable B41 background, the binding of bnAbs PGT145, PG16, VRC01, 35O22, and PGT121 were even less altered by V4.2 mutations (Figure 8C and Tables S2–S4). nAb b12, and the V3 tip-specific Abs likewise were less affected by v4.2 stabilization in B41 with similar affinities of 3869 and 447-52D, whereas binding of 3074 was not detectable in the stabilized construct. The V2i Ab 2158 was unable to bind B41, and the affinity of 830A was comparable between B41.664 and v4.2. These results indicate that for trimers such as B41 that are already relatively fixed in the pre-fusion conformation, the additional SOSIPv4.2 modifications do not have major local or global impacts on structural ordering or antigenicity.

As with AMC008 and B41, binding of bnAbs PGT145, PG16, VRC01, 35O22, and PGT121 were relatively unaffected comparing BG505.664 and BG505V4.2. Unexpectedly, however, b12 affinity for BG505 increased for the v4.2 construct. It has been shown via neutralization assays that alanine amino acid substitutions in the V3 loop at locations near the A316W mutation in the v4 constructs (such as F317A and Y318A in the crown of V3) can increase the neutralization potency of b12 for pseudo-typed virus, but a structure-based explanation for this phenomenon is not available at present (Falkowska et al., 2012).

All three V3 tip binding Abs showed abrogated binding to the BG505v4.2 construct. The attenuated binding was largely driven by a decrease in k_on_, with little change in k_off_, which suggests the weakened V3 tip binding is likely owing to decreased epitope accessibility (Tables S2–S4). Alternatively, the A316W mutation is adjacent to direct binding contact residues for 447-52D, 3074, and 3869; thus, it is possible the mutation influences the binding of these antibodies. However, this may be less likely for 3869 and 447-52D because the A316W mutation did not alter the binding kinetics in B41. BLI reported weak binding for 830A and 2158 against BG505.664, but there was no measurable signal in the v4.2 construct using similarly high concentrations. We note that HDX-MS did not show a difference in deuterium exchange for these regions resulting from the SOSIP.v4 mutations, thus reflecting a limitation of the method for capturing some specific differences in structure that are present in SOSIP.664 and v4 trimers.

In summary, a comparison of these three isolates against their stabilized counterparts suggests strains that are more inherently flexible, such as AMC008, may be more susceptible to attenuating the binding of antibodies specific for the open conformation, the tip of V3, and parts of the V2 loop through mutation-induced conformational fixation. In addition, the potent bnAbs that have the greatest documented neutralization breadth among the antibodies we examined were only modestly impacted in their binding, perhaps reflecting their impressive cross-reactivity and ability to tolerate extreme levels of structural and sequence variation in their cognate antigen targets.

## Discussion

HIV Env exhibits tremendous genetic sequence diversity as a result of its rapid mutation rate and intense selective immune pressures. Here we sought to identify the structural and dynamic manifestations of sequence diversity, adding an important dimension to our understanding of HIV Env as an antigenic target that complements available static high-resolution information. Isolate-specific differences in local epitope organization and conformational equilibria have been challenging to characterize in the past. Indeed, structures of Env are most often determined with stabilizing antibody fragments bound and are the end result of stringent selection of sub-populations of particles in single particle cryo-EM analysis and conformational fixation in X-ray crystallography. These factors lock in conformational states and suppress protein dynamics. Biophysical techniques such as smFRET and DEER spectroscopy show dynamic motions and conformational heterogeneity but are limited in resolution, and, at present, a clear assignment of spectroscopically defined sm-FRET states to Env structures remains ambiguous (Pan et al., 2020; Stadtmueller et al., 2018).

Here we used HDX-MS to bridge structure and dynamics in the Env trimer and show that substantial differences in local structural dynamics are evident in Env derived from five HIV isolates. The results of this study provide the first detailed analysis of structural variation among divergent HIV Env trimers and reveal structural properties such as regional conformational switching and differences in the local epitope order.

We observed peptides indicative of conformational switching in regions previously shown to map to two distinct allosteric networks upon CD4 binding (Guttman et al., 2014) (Figure 6D). In this case, however, the local structural changes are occurring in the absence of CD4. This suggests that the trimers are in equilibrium between the closed pre-fusion and a more open conformation that is largely consistent with a CD4 receptor-bound conformation. These transitions occur across timescales ranging from seconds to hours depending on the isolate and allow us to compare the relative rates of Env opening motions across distinct isolates. Furthermore, the distinct timescales for different regions suggest that these motions are not concerted across the trimer; instead, a degree of modularity appears to govern conformational dynamics. Widely separated sites such as V3, HR1, and HR2 peptides sample distinct conformations on the timescale of seconds, whereas the bridging sheet and gp120 inner domain helix undergo much less frequent conformational transitions with kinetics measured in hours.

It is interesting to speculate about the extent to which glycans may play a role in strain-specific differences in dynamics and conformation. Glycosylation has been suggested to influence dynamics in Env and more recently in the SARS-CoV2 spike protein (Sztain et al., 2021). For example, loss of the N301 glycan seems to increase V3 loop flexibility (Powell et al., 2017), and loss of the N160 glycan has been posited to increase the V1V2 loop flexibility in turn destabilizing the V3 pocket making it easier for V3 crown targeting antibodies to bind (Andrabi et al., 2015; Lee et al., 2017a). From our mass spectrometry data, we did a relative isolate comparison of the glycan occupancy and glycan heterogeneity using different proteases (Table S5). The majority of glycosylation differences were in the highly variable V1 loop (which explains the missing coverage in the HDX-MS data in this region), but heterogenous glycoforms were present across all isolates at N301 and N160 (although we did not have coverage here in JR-FL). From our glycoprofiling data, we did not observe any correlations between glycosylation and conformational switching that were immediately recognizable.

Our results shed light on a structural model that has been proposed to explain the basis for neutralization resistant phenotypes (deCamp et al., 2014; Guttman et al., 2015; Montefiori et al., 2018). In this model Env from neutralization resistant, isolates adopt a closed pre-fusion conformation, whereas sensitive isolates sample an open conformation more readily. From our analysis, surprisingly, the tier 2 CE1176 isolate exhibited a highly dynamic phenotype similar to AMC008. However, unlike AMC008, we observed that CE1176 has, in fact, the most highly protected V3 loop (Figure 3). The neutralization-sensitive AMC008 isolate, by contrast, exhibits a relatively dynamic V3 loop. These data may thus help explain the neutralization resistant tier 2 assignment of CE1176 and neutralization sensitive designation for AMC008 using pooled human serum from infected donors that is rich in V3-specific antibodies (Han et al., 2019; Hraber et al., 2018; Montefiori et al., 2018). Isolate-specific differences in local epitope flexibility and global conformational switching in Env trimers may help provide a structure-based explanation for differences in antigenicity and neutralization sensitivities in addition to the more immediately recognizable differences in glycosylation and sequence variation. Further analysis of diverse HIV isolates is warranted to test the hypothesis that neutralization sensitivity is directly correlated with Env open/closed equilibria (deCamp et al., 2014; Seaman et al., 2010).

These differences in epitope rigidity likely contribute to differences in antigenicity between isolates, but variability in Env sequences and glycosylation are clearly important contributing factors as well. By examining the effect of conformational fixation in a given Env background, we have been able to characterize the impact on dynamics and antigenicity. Our data suggest that the stabilizing v4 SOSIP mutations can suppress dynamics but appear more effective when applied to conformationally dynamic Env trimers such as AMC008. The effect of the SOSIPv4 mutations depends on the background Env to which it is introduced as reflected by the muted effect observed by HDX-MS comparisons of B41 and BG505 SOSIP.664 and v4 trimers. The lack of any change in dynamics in B41 after the introduction of the v4 mutations could be because B41 was generally more structurally fixed than the other four isolates, including a region spanning the E64K/H66R mutation sites meant to lock Env in the closed state (Figure 3, peptide 10). Increased protection in the gp120 layer 1 suggests B41 may more stably maintain the short helix seen in many gp120 core crystal structures (Kwon et al., 2012). The antigenic effects were observed most potently in reduction in non-neutralizing antibody binding, which is critical for reducing immune responses against immunodominant but non-protective epitopes. Surprisingly, however, mature bnAbs in many cases only exhibited modest increases in binding to the more stabilized forms of the trimers. Perhaps, over the course of their development, they evolved to tolerate extreme levels of structural and dynamic variation in their targets, much as they evolved to recognize conserved residues while skirting sites that vary among isolates.

Beyond v4 SOSIPs, other hyperstabilized SOSIPs and Env constructs have been designed to further abrogate the inherent trimer flexibility (de la Peña et al., 2017; Georgiev et al., 2015; Rawi et al., 2020; Torrents de la Pena and Sanders, 2018; Ward and Wilson, 2017). This strategy of stabilizing the pre-fusion protein structure, which is the primary target for nAbs, originated in the HIV Env SOSIP design, but has since been adopted for stabilization of pre-fusion conformations in respiratory syncytial virus (McLellan et al., 2013) and SARS-CoV-2 spike trimers (Hsieh et al., 2020a; Wrapp et al., 2020) where the improvement in potent neutralization activity appears directly a result of the fixation of the pre-fusion conformations (Sanders and Moore, 2021). As is now appreciated, however, in the case of a hypervariable antigenic target such as HIV Env, achieving neutralization breadth requires not only maintaining a pre-fusion conformation for the viral protein, but also training the immune system to focus on conserved epitopes while avoiding potentially immunodominant, distracting isolate-specific epitopes (Derking and Sanders, 2021; D'Souza et al., 2020).

A better understanding of how sequence diversity affects the structural dynamic behavior of Env provides a foundation to better understand structural correlates of antibody neutralization of HIV. Lastly, an understanding of the dynamic antigenic target and how structure-based modifications modulate dynamics and epitope exposure can help guide future Env-based HIV vaccine designs.

### Limitations of the study

HDX-MS was examined from 3 s to 20 h. Faster dynamic events that may be occurring within the first 3 s or after 20 h are not sampled. Large differences in protein sequence also reduce the number of homologous peptides with the same number of exchangeable amides that can be directly compared. Gaps in protein coverage (coverage ∼60%) limit which regions can be observed across isolates, leaving some sites that may behave differently that are not being monitored. In the future, coverage could be improved by using additional proteases that are active at the low pH required for HDX-MS analysis, though the problem of dense heterogenous glycosylation remains, reducing some sequence coverage. We also note it has been demonstrated that some backbone amide exchange cannot be accounted for via solvent accessibility or secondary structure alone via experiments using HDX-MS and molecular dynamics simulations (McAllister and Konermann, 2015).

## STAR★Methods

### Key resources table

**Table:** 

REAGENT or RESOURCE	SOURCE	IDENTIFIER
**Antibodies**		

Monoclonal anti-HIV-1 Env VRC01	https://aidsreagent.org/	Cat# 12033;RRID:AB_2491019
Monoclonal anti-HIV-1 Env 35O22	This Study, (Huang et al., 2014)	N/A
Monoclonal anti-HIV-1 Env PGT145	This Study, (Walker et al., 2011)	N/A
Monoclonal anti-HIV-1 Env PG16	https://aidsreagent.org/	Cat# 12150; RRID:AB_2491031
Monoclonal anti-HIV-1 Env b12	This Study, (Burton, 1991)	N/A
Monoclonal anti-HIV-1 Env 17b	This Study, (Kwong et al., 1998)	N/A
Monoclonal anti-HIV-1 Env PGT121	This Study, (Walker et al., 2011)	N/A
Monoclonal anti-HIV-1 Env 447-52D	Laboratory of Susan Zolla-Pazner (Upadhyay et al., 2014)	N/A
Monoclonal anti-HIV-1 Env 3074	Laboratory of Susan Zolla-Pazner (Upadhyay et al., 2014)	N/A
Monoclonal anti-HIV-1 Env 3869	Laboratory of Susan Zolla-Pazner (Upadhyay et al., 2014)	N/A
Monoclonal anti-HIV-1 Env 830A	Laboratory of Susan Zolla-Pazner (Upadhyay et al., 2014)	N/A
Monoclonal anti-HIV-1 Env 2158	Laboratory of Susan Zolla-Pazner (Upadhyay et al., 2014)	N/A

**Chemicals, peptides, and recombinant proteins**		

Pro-Pro-Pro-Phe (PPPF)	Anaspec	N/A
Deuterium oxide 99.96%	Cambridge Isotope Laboratories	Cat# DLM-6-10X0.75
25 kDa Polyethylenimine (PEI)	Polysciences	Cat# 23,966
Dulbecco’s Modified Eagle Medium (DMEM)	ThermoFisher	Cat# 10313-021
Fetal Bovine Serum (FBS)	ThermoFisher	Cat# 10437-028
Freestyle 293 Expression Medium	ThermoFisher	Cat# 12338-018
Galanthus nivalis lectin-agarose	Vector Laboratories	Cat# AL-1243-5
Methyl α-D-mannopyranoside	Millipore Sigma	Cat# M6882-25G
Amicon Ultra-4, MWCO 100kDa	Millipore Sigma	Cat# UFC910024
Amicon® Ultra-15 Centrifugal Filter Unit, 30KDa, 24	Millipore	Cat# UFC903024
Slide-A-Lyzer™ Dialysis Cassettes, 20K MWCO	ThermoScientific	Cat# 66030
Pierce™ Protein A IgG Purification Kit, 1 mL	ThermoScientific	Cat# 44667
NuPAGE™, 4–12% Bis-Tris Protein Gels	ThermoScientific	Cat# NP0321BOX
NativeMark™ Unstained Protein Standard	ThermoScientific	Cat# LC0725
NativePAGE™ 20X Running Buffer	ThermoScientific	Cat# BN2001
Tween 20	Millipore Sigma	Cat# P7949
HiTrap DEAE Fast Flow	Millipore Sigma	Cat# GE17-5055
HiTrap Phenyl	Millipore Sigma	Cat# 17-5195
HiLoad 16/600 Superdex 200 pg	Millipore Sigma	Cat# GE28-9893-35
BG505.SOSIP.664	This Study	N/A
BG505.SOSIPv4.1	This Study	N/A
BG505.SOSIPv4.2	This Study	N/A
B41.SOSIP.664	This Study	N/A
B41.SOSIPv4.1	This Study	N/A
B41.SOSIPv4.2	This Study	N/A
AMC008.SOSIP.664	This Study	N/A
AMC008.SOSIPv4.1	This Study	N/A
AMC008.SOSIPv4.2	This Study	N/A
JRFL.SOSIP.664	This Study	N/A
CE1176.SOSIP.664	This Study	N/A

**Experimental models: Cell lines**		

Freestyle293F	Thermo-Fisher Scientific	Cat# R79007

**Recombinant DNA**		

BG505.SOSIP.664	(Sanders et al., 2013b)	N/A
BG505.SOSIPv4.1	(de Taeye et al., 2015)	N/A
BG505.SOSIPv4.2	(de Taeye et al., 2015)	N/A
B41.SOSIP.664	(de Taeye et al., 2015)	N/A
B41.SOSIPv4.1	(de Taeye et al., 2015)	N/A
B41.SOSIPv4.2	(de Taeye et al., 2015)	N/A
AMC008.SOSIP.664	(de Taeye et al., 2015)	N/A
AMC008.SOSIPv4.1	(de Taeye et al., 2015)	N/A
AMC008.SOSIPv4.2	(de Taeye et al., 2015)	N/A
JRFL.SOSIP.664	This Study	N/A
CE1176.SOSIP.664	This Study	N/A

**Software and algorithms**		

UCSF ChimeraX	UCSF	https://www.rbvi.ucsf.edu/chimerax/
ImageJ	(Schneider et al., 2012)	https://imagej.nih.gov/ij/
ForteBio Octet Data Analysis v11 software	ForteBio	N/A
ForteBio Data Acquisition v11	ForteBio	N/A
HDExaminer v2	Sierra Analytics	http://massspec.com/hdexaminer/
DriftScope	Waters	https://www.waters.com/nextgen/us/en.html
Byonic	Protein Metrics	https://proteinmetrics.com/byos/
HX-Express v2	(Guttman et al., 2013)	N/A

**Other**		

Anti-human Fc capture (AHC) Biosensors	ForteBio	Cat# 18-5060
Synapt G2-Si Q-TOF mass spectrometer	Waters	N/A
Orbitrap Fusion Tribrid Mass Spectrometer	ThermoScientific	N/A
Octet RED96 System	ForteBio	N/A

### Resource availability

#### Lead contact

Further information and requests for resources and reagents should be directed to and will be fulfilled by the lead contact, Prof Kelly Lee (kklee@uw.edu).

#### Materials availability

This study did not generate new unique reagents.

### Experimental models and subject details

#### Cell lines for antibody production

HEK293F cells (ThermoFisher Scientific) used for expression of antibodies were grown at 37°C, 8% CO2, with shaking at 125 rpm in Freestyle 293 media (ThermoFisher Scientific) without additives. The cell lines were not authenticated within our lab, however a certificate of analysis from the manufacturer is available. The gender of the 293F cell line is female.

#### Cell lines for SOSIP production

HEK293F cells (ThermoFisher Scientific) used for expression of SOSIP Env glycoproteins were grown at 37°C, 8% CO2, with shaking at 125 rpm in Freestyle 293 media (ThermoFisher Scientific) without additives. The cell lines were not authenticated within our lab, however a certificate of analysis from the manufacturer is available. The gender of the HEK293F cell line is female.

### Method details

#### Protein expression and purification

SOSIPs were produced and purified as previously described by Verkerke et al. and briefly below. The JR-FL SOSIP was kindly provided by the Shiu-Lok Hu lab. Briefly, HEK293F cells were transiently transfected at a density between 0.8 and 1.2 million cells/mL using polyethylenimine (PEI) with plasmids encoding each SOSIP isolate co-transfected with furin in pcDNA3.1 at a ratio of three SOSIPs to one furin to ensure proteolytic cleavage between gp120 and gp41 subunits during production. After roughly six days the cell supernatants were cleared by centrifugation and filtered through a 0.2-micron vacuum filtration unit and supplemented with protease inhibitors (Roche) and sodium azide to prevent microbial growth. Glycosylated trimers were extracted using *Galanthus nivalis* lectin (GNL) coupled to agarose beads overnight at 4°C and washed with 20 mM Tris (pH 7.4), 1 mMEDTA, 1 mM EGTA, 0.02% azide, and 120 mM NaCl; glycoproteins were eluted with 7–10 column volumes of 1 M alphamethyl-mannopyrannoside dissolved in 20 mM Tris (pH 7.4), 1 mM EDTA, 1 mM EGTA, 0.02% azide, and 120 mM NaCl. GNL eluates were concentrated using Amicon ultrafiltration units (nominal molecular mass cutoff of 100 kDa) and buffer exchanged into DEAE low-salt buffer (20 mM Tris [pH 8.0], 100 mM NaCl) before anion-exchange chromatography using a DEAE column. Following 10 min of isocratic flow in 100 mM NaCl, a gradient to 1 M NaCl was initiated and fractions were collected through-out to remove protein aggregates. The DEAE flowthrough was buffer exchanged into 2 M ammonium sulfate– 0.1 M phosphate (pH 7.4) via dialysis and loaded onto a 5 mL HIC HiTrap Phenyl HP column. A step-wise gradient of 2M to 0 M ammonium sulfate in 0.1 M phosphate (pH 7.4) over 90 min was used to separate trimers from dimers and monomers. The early-eluting fractions (containing native-like trimers) were concentrated prior to being loaded onto a Superdex S200PG size exclusion chromatography (SEC) column in PBS (20 mM sodium phosphate [pH 7.4], 150 mM sodium chloride, 0.02% sodium azide). Peak fractions were concentrated and characterized by DLS, SDS, BN-PAGE, and negative stain EM (Figure S3) to ensure homogenous, pure trimer populations immediately prior to HDX and BLI experiments.

The V2i and V3 tip IgG Abs were kindly provided by Susan Zolla-Pazner for BLI experiments. VRC01, and PG16 were provided by NIH AIDS Research and Reference Reagent Program. The other antibodies were produced by transient co-transfection of plasmids containing heavy and light chain fragments at a 1:1 ratio in HEK293F cells. Cultures were allowed to grow for approximately 6 days before harvesting. Secreted IgG was isolated and purified by affinity chromatography using a Hi-Trap Protein A column and eluted using 100 mM Glycine pH2.0 and neutralized by addition of 1 M Tris pH8.0. Purity was assessed by SDS-PAGE.

#### SDS-PAGE and BN-PAGE

SDS denaturing PAGE and blue native PAGE (BN-PAGE) analyses with precast gels (Novex) were performed to assess the oligomeric species present throughout purification and immediately prior to experiments. Typically, between 10 and 15 μg of protein was loaded per lane for BN-PAGE analysis and 5 μg per lane was loaded for SDS-PAGE analysis.

#### Dynamic light scattering (DLS)

Dynamic light scattering (DLS) measurements were performed on a Dynapro Nanostar (Wyatt Technologies). Trimer samples were diluted to 1 mg/mL in PBS and centrifuged at 15,000xg for 20 min prior to loading of 10 μL into a low-volume quartz cuvette. The mean estimated hydrodynamic radius, and polydispersity were generated from 30 acquisitions of 5 s at 20°C.

#### Hydrogen/deuterium exchange mass spectrometry

5 μgs (42 pmol) per timepoint of each protein were incubated in deuterated buffer (20 mM PBS, 85% D2O, pH∗7.52) for 3 s, 1 min, 30 min, and 20 hrs at room temperature. The reaction was stopped via diluting 1:1 in ice-cold quench buffer (200 mM tris(2-chlorethyl) phosphate (TCEP), 8 M urea, 0.2% formic acid) to a final pH of 2.5 and flash frozen in liquid nitrogen followed by storage in −80°C prior to analysis. Online pepsin digestion was performed and analyzed by LC-MS-IMS utilizing a Waters Synapt G2-Si Q-TOF mass spectrometer as described previously utilizing a 15 min gradient and a home-made HDX cold box that maintains the pepsin digestion at 4°C and the LC plumbing at 0°C (Verkerke et al., 2016; Watson et al., 2021). Pepsin digest eluates from undeuterated sample LC-MS runs were collected, dried by speed vac, incubated in deuteration buffer for 1 h at 65°C, and quenched as described above to prepare fully deuterated controls. Pepsin digest eluates from undeuterated sample LC-MS runs were also collected, dried by speed vac, resuspended in mobile buffer for peptide identification using nano LC-MS on an Orbitrap Fusion mass spectrometer. A 2 cm trapping column and a 35 cm analytical column were freshly prepared in fused silica (100 μm ID) with 5 μM ReproSil-Pur C18 AQ beads (Dr. Maisch). 8 μL of sample was injected and run using a 60-min linear gradient from 2% to 30% acetonitrile in 0.1% FA, followed by 10 min of 80% acetonitrile. An EThcD method was optimized as follows: ion source: 2.1 kV for positive mode; ion transfer tube temperature: 350°C; resolution: MS1 = 120000, MS2 = 30000; AGC target: MS1 = 2e5, MS2 = 1e5; and injection time: MS1 = 50 ms, MS2 = 60 ms. Orbitrap Fusion data was processed using Byonic (Version 3.8, Protein Metrics Inc.) to obtain a peptide reference list and identify peptic glycopeptides and glycosylation sites. Deuterium uptake analysis was performed with HD-Examiner (Version 2.5, Sierra Analytics) followed by HX-Express v2 for binomial fitting (Guttman et al., 2013; Weis et al., 2006). The percent exchange was normalized to the fully deuterated samples. The well characterized BG505 SOSIP was exchanged alongside each new construct as a positive control sample and Internal exchange standards (Pro-Pro-Pro-Ile [PPPI] and Pro-Pro-Pro-Phe [PPPF]) were included in each reaction to control for variations in ambient temperature during the labeling reactions. For isolates that were exchanged independently, the differences in %deuteration of each homologous peptide with the same number of exchangeable amides were only considered significant if the differences were greater than the variability observed in that homologous peptide across the BG505 controls on a peptide by peptide basis.

#### Biolayer interferometry

The binding kinetics of the SOSIP constructs against a panel of IgG’s were determined via BLI on an Octet Red system (FortèBio). Anti-human IgG Fc capture biosensors were presoaked in binding buffer (phosphate-buffered saline (PBS pH 7.4) supplemented with 0.1% BSA, 0.005% Tween 20, and 0.02% NaN3) for 10 min. The hydrated tips were then loaded with purified IgG prepared at 8 μg/mL in binding buffer for 80 s. After reaching a stable baseline, antibody-immobilized biosensors were moved into wells containing a 2-fold dilution series of SOSIP trimer to monitor association for 3 min, then biosensors were moved back into wells containing binding buffer to monitor dissociation for 3 min. Responses were calculated and double referenced against the buffer reference signal and non-specific binding of analyte to biosensor in absence of IgG. Kinetic data were analyzed by using FortéBio Data Analysis 11.0 software and were processed by Savitzky-Golay filtering prior to fitting using a 1:1 binding model. Reported values are averages of data repeated in at least two independent experiments.

#### Glycan profiling

To identify the occupancy of glycans at each glycosite and assess the relative heterogeneity of glycoforms at each site bottom up mass spectrometry (MS) was utilized. SOSIPs (0.02mgs) were denatured in a solution containing 25 mM Tris (pH 8.0), 7 M guanidinium chloride (GdnHCl) and 50 mM dithiothreitol (DTT) at 90°C for 30 min. Reduced cysteines were alkylated by adding fresh iodoacetamide (IAA) to 100 mM and incubating at room temperature for 1 h in the dark. 50 mM excess DTT was then added to quench the remaining IAA. The GndHCl concentration was reduced to 0.6 M by diluting the samples 11-fold with a 10 mM Tris (pH 8.0), 2 mM calcium chloride solution. Samples were then digested using trypsin, and chymotrypsin separately at a ratio of 1:30 (w/w) for 4 h at 37°C, or a combination of Lys-C and Glu-C at a ratio of 1:30 (w/w). Each SOSIP was digested first by Lys-C for 4 h at 37°C followed by an overnight digestion of Glu-C 37°C. All proteases were MS grade (Promega) and the digestion reactions were quenched by the addition of 0.02% formic acid. The digested samples were desalted by Sep-Pak C18 cartridges (Waters) following the manufacturer’s suggested protocol. Glycoform determination was performed via nano LC-MS using an Orbitrap Fusion mass spectrometer (Thermo Fisher) as described above in the HDX-MS method section using EThcD and the processing software Byonic (Version 3.8, Protein Metrics Inc.) using a 6 ppm precursor and 10 ppm fragment mass tolerance. Glycopeptides were searched using the N-glycan 309 mammalian database in Protein Metrics PMI-Suite and scored based on the assignment of correct c- and z- fragment ions. The true-positive entities were further validated by the presence of glycan oxonium ions m/z at 204 (HexNAc ions) and 366 (HexNAcHex ions). Glycoforms were categorized as either high mannose: HexNAc(2)Hex(9-5); Hybrid: HexNAc(3)Hex(5-6); or complex (Table S5).

### Quantification and statistical analysis

All of the statistical details of experiments can be found in the Figure legends of each Figure. Hydrogen/deuterium-exchange mass spectrometry experiments were performed in triplicate. Deuterium uptake levels shown in Figures 3, 4, 7, S3, S4, S11B, S12C, S13C, S14B, and S15B were determined using HD-Examiner (Sierra Analytics) with the error bars reflecting the standard deviation of the technical replicates. Deuterium uptake levels shown in S2 compare BG505 control biological replicates and error bars reflect the standard deviation of technical replicates. Mass spectral analysis to test for the presence of bimodal spectra was performed with HX-Express v2 (Weis et al., 2006; Guttman et al., 2013) and error bars in the population bar plots seen in Figure 6 reflect the standard deviation in % population abundance of the technical replicates.

Biolayer interferometry (BLI) experiments were carried out in at least duplicate. Data represent mean ± the standard deviation as indicated in the Figure legends.

## Data Availability

• The raw HDX data is is provided in the supplemental Data S1 and will be shared by the lead contact upon request. • The study did not generate/analyze any dataset/code and does not report any original code. • Any additional information required to reanalyze the data reported in this paper is available from the lead contact upon request.

## Supplemental information

Document S1. Figures S1–S8 and Tables S1–S4

Table S5. Isolate specific differences in glycan occupancy and glycan heterogeneity, related to STAR Methods The attached excel sheet lists each isolate in a separate worksheet. Each Asn (N) residue with the NxS/T motif indicating a glycosite is in a column with a list of the glycoforms identified via Ms/Ms using EThcD. Regions with no coverage are listed as NC, and ND indicates areas where the glycoform was not determined (for example low quality/scoring Ms/Ms spectra). Glycoforms were categorized as either high mannose: HexNAc(2)Hex(9-5) (green); Hybrid: HexNAc(3)Hex(5-6) (blue); or complex (red). Abbreviations and glycan classifications used are according to Essentials of Glycobiology, 3rd ed. HexNAc: N-acetylhexosamines; Hex: Hexose; Fuc: L-Fucose; NeuAc: N-acetyl neuraminic acid.

Data S1. HDX Uptake Plots, related to Figures 2 and 7 The attached excel sheet lists each isolate and stabilized construct in a separate worksheet. The HDX uptake plots are shown and each data point is the average % deuteration at each time point (3 s, 1 min, 30 min, and 20 h) normalized to a fully deuterated control with standard deviation bars from at least two replicates. JRFL.664 uptake curves are colored purple and CE1176.664 uptake curves are colored orange. For BG505, B41, and AMC008 the SOSIP.664 uptake curves are colored blue, SOSIPv4.1 uptake curves are colored green and SOSIPv4.2 uptake curves are colored red. Peptides spanning the E64K, H66R, A316W, I535 M, and L543N mutation sites are displayed as X
