# Supplementary material for: Structural dynamics reveal isolate-specific differences at neutralization epitopes on HIV Env
Source: iScience. 2022 May 23;25(6):104449. doi: 10.1016/j.isci.2022.104449 (PMC9167985; doi:10.1016/j.isci.2022.104449)
Supplement: Document S1. Figures S1–S8 and Tables S1–S4 [file mmc1.pdf]

iScience, Volume *25*

## Supplemental information

# Structural dynamics reveal isolate-specific differences at neutralization epitopes on HIV Env

**Edgar A. Hodge, Gajendra S. Naika, Sally M. Kephart, Adam Nguyen, Richard Zhu, Mark A. Benhaim, Wenjin Guo, John P. Moore, Shiu-Lok Hu, Rogier W. Sanders, and Kelly K. Lee**

## Supplemental Figures.

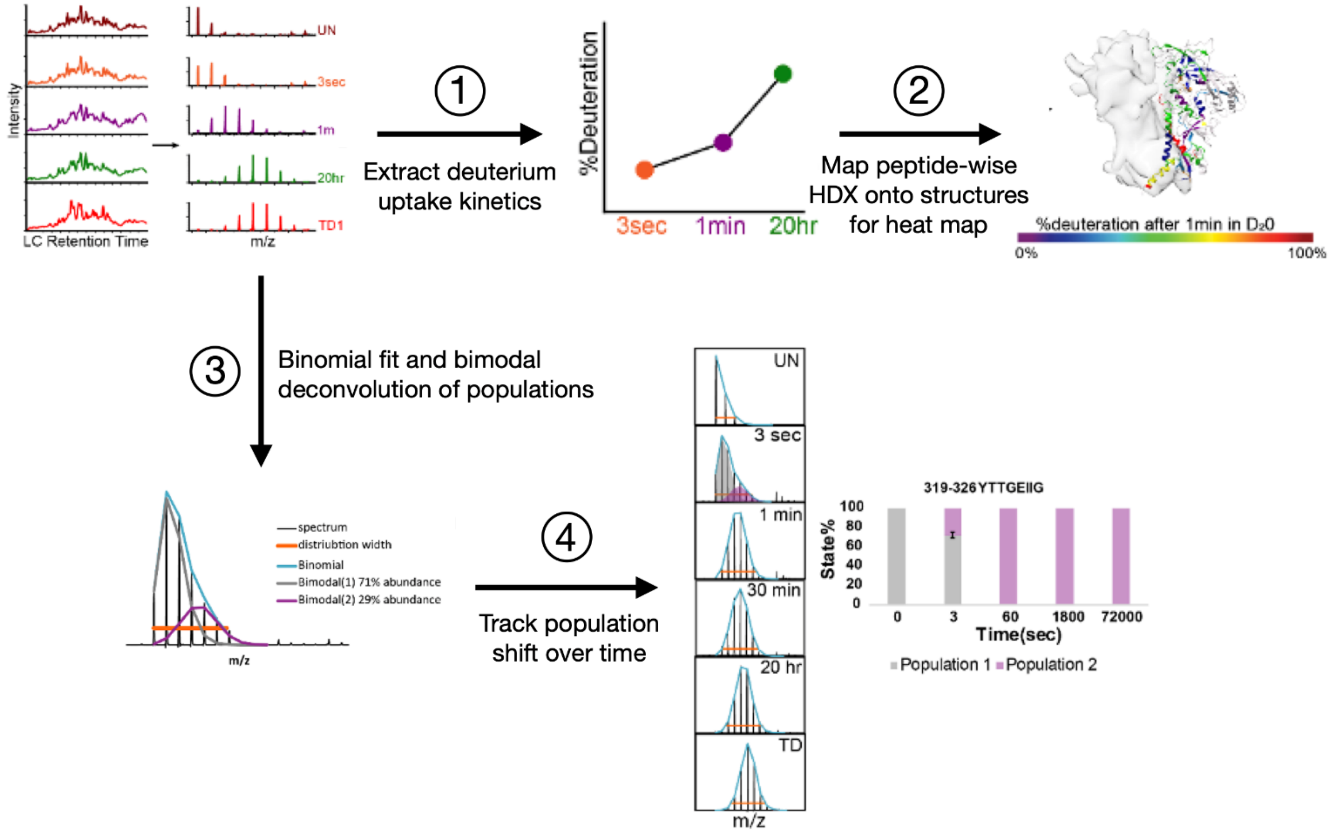


**Figure S1. HDX analysis workflow, related to Figures 1-7**

Initially after data collection each peptide's m/z, charge state, unique retention time and drift time are uploaded into HDExaminer, which extracts each peptide's mass spectra at each time point (1) and plots the % deuteration with time based on centroid fitting. The percent deuteration of each peptide at the 1minute timepoint is then color coded and mapped onto the structure of a SOSIP (2) to generate an HDX heatmap for each individual isolate. Next the HDExaminer centroid fit data is exported into HX-Expressv2 for binomial fitting (3). The analysis in HX-Express takes into account the mass envelope distribution width. Anything unusually broad is flagged as bimodal, and bimodal deconvolution allows for the identification of EX1 kinetics that are indicative of conformational switching. The percent abundance of the two populations (colored gray and purple respectively) across time are then converted into a bar plot (4) to determine the rate of the conformational transition from the protected to the exposed populations.

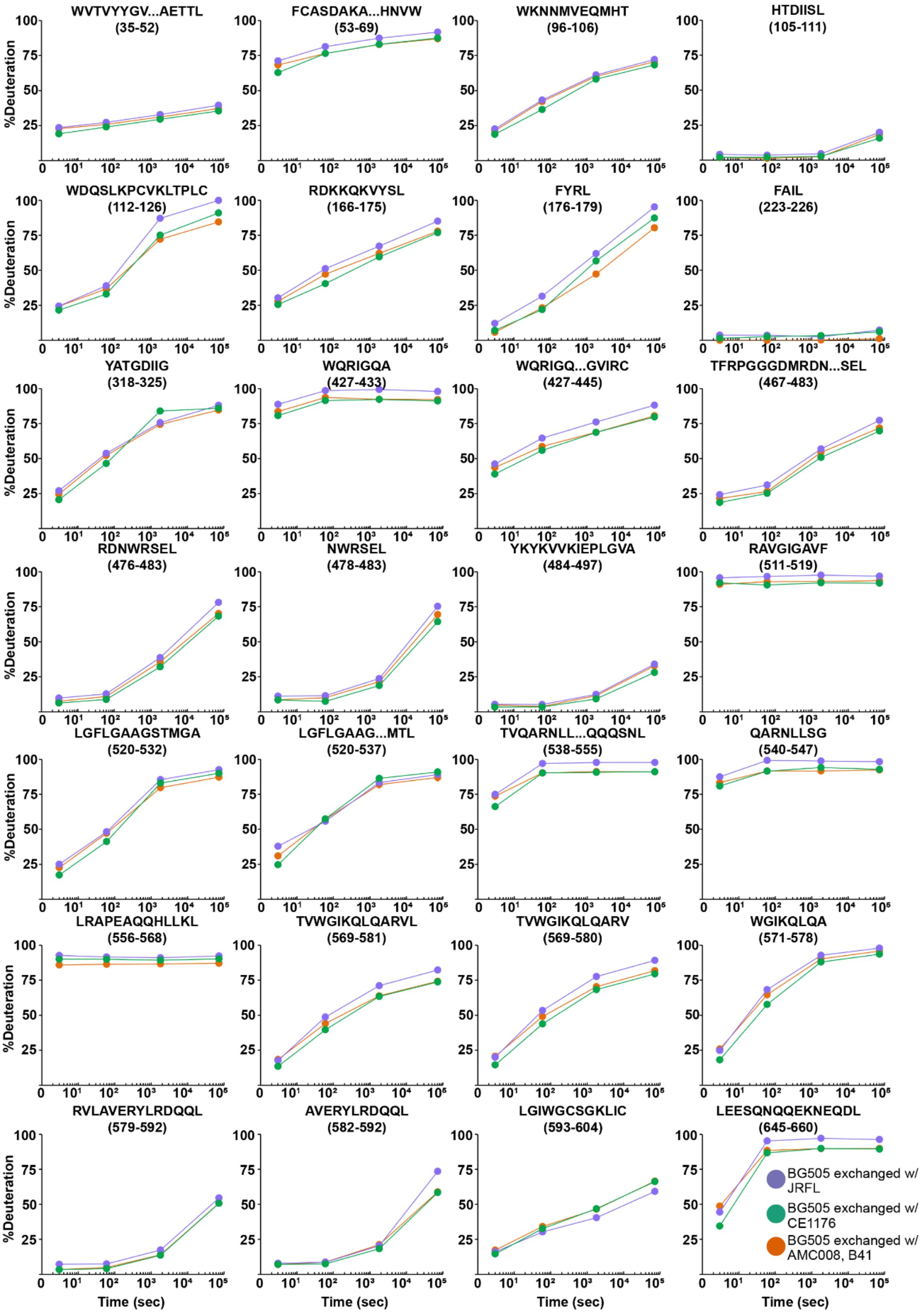

WVTVYYGV...AETTL (35-52)
FCASDAKA...HNVW (53-69)
WKNNMVEQMHT (96-106)
HTDIISL (105-111)
WDQSLKPCVKLTPLC (112-126)
RDKKQKVYSL (166-175)
FYRL (176-179)
FAIL (223-226)
YATGDIIG (318-325)
WQRIGQA (427-433)
WQRIGQ...GVIRC (427-445)
TFRPGGGDMRDN...SEL (467-483)
RDNWRSEL (476-483)
NWRSEL (478-483)
YKYKVVKIEPLGVA (484-497)
RAVGIGAVF (511-519)
LGFLGAAGSTMGA (520-532)
LGFLGAAG...MTL (520-537)
TVQARNLL...QQQSNL (538-555)
QARNLLSG (540-547)
LRAPEAQQHLLKL (556-568)
TVWGIKQLQARVL (569-581)
TVWGIKQLQARV (569-580)
WGIKQLQA (571-578)
RVLAVERYLRDQQL (579-592)
AVERYLRDQQL (582-592)
LGIWGCSGKLIC (593-604)
LEESQNQQEKNEQDL (645-660)
%Deuteration
Time (sec)
BG505 exchanged w/ JRFL
BG505 exchanged w/ CE1176
BG505 exchanged w/ AMC008, B41

**Figure S2. BG505 HDX reference controls, related to Figures 3-4**

Percent deuteration of commonly found homologous peptides across different isolates from BG505 biological replicates that were exchanged alongside other isolates as an HDX control standard. Purple uptake curves represent BG505 exchanged alongside JRFL, green was BG505 exchanged in tandem with CE1176, and orange indicates BG505 exchanged in tandem with AMC008 and B41 constructs. Each time point is the average of duplicate measurements with error bars displaying standard deviation.

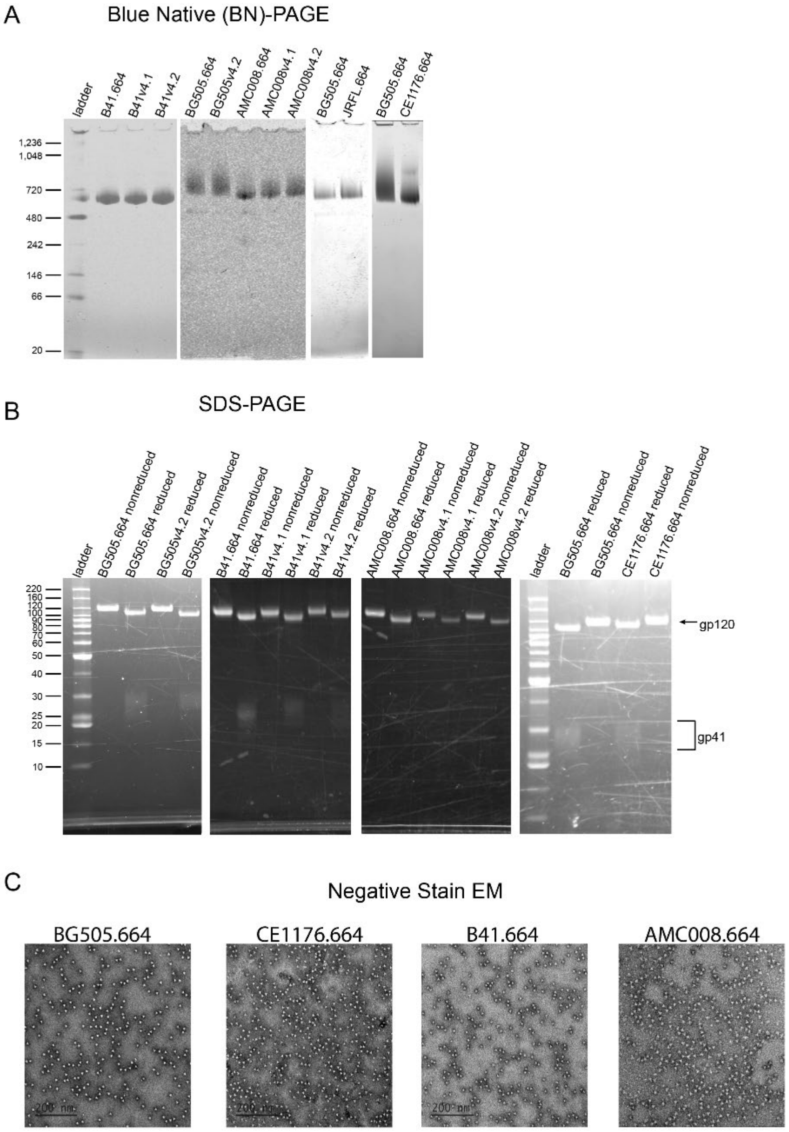


D

| SOSIP | Avg radius (nm) | Radius std dev | Avg %polydispersity | Avg %abundance |
|---|---|---|---|---|
| BG505.664 | 6.7 | 0.10 | 9.0 | 100 |
| BG505v4.2 | 6.9 | 0.11 | 11.4 | 100 |
| B41.664 | 6.8 | 0.21 | 12.9 | 100 |
| B41v4.1 | 7.0 | 0.12 | 14.5 | 100 |
| B41v4.2 | 6.8 | 0.1 | 15.8 | 100 |
| AMC008.664 | 7.0 | 0.1 | 10.6 | 100 |
| AMC008v4.1 | 7.0 | 0 | 13.7 | 100 |
| AMC008v4.2 | 7.0 | 0 | 16.6 | 100 |
| JRFL.664 | 7.0 | 0.06 | 12.9 | 100 |
| CE1176.664 | 7.0 | 0.07 | 13.1 | 100 |

**Figure S3. SOSIP characterization by blue native (BN)-PAGE, SDS-PAGE, and dynamic light scattering (DLS), related to STAR Methods.**

To assess trimer purity each SOSIP was characterized by BN-PAGE (A), SDS-PAGE (B), negative stain EM (C), and DLS (D) after purification and prior to HDX and BLI experiments. On the SDS-PAGE each sample was run non-reduced and reduced via addition of DTT. The single band in the non-reduced lane runs at a slightly higher MW due to the presence of an additional inter-subunit disulfide bond linking gp120 and gp41. The weakly visible gp41 bands in the reduced lane are smeared likely due to glycan heterogeneity.

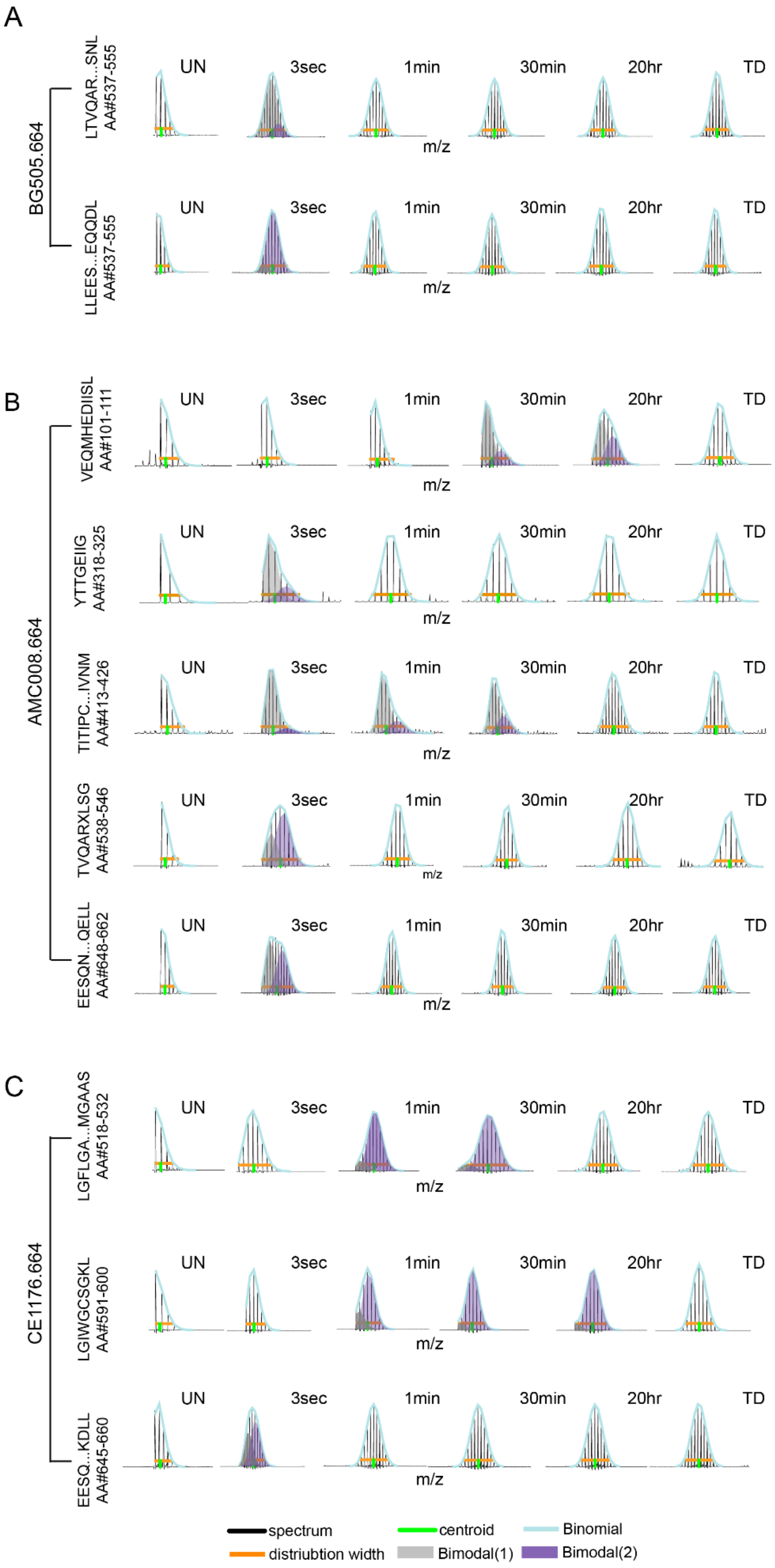
A
B
C
BG505.664
AMC008.664
CE1176.664
LTVQAR...SNL AA#537-555
LLEES...EQQDL AA#537-555
VEQMHEDIISL AA#101-111
YTTGEIIG AA#318-325
TITIPC...IVNM AA#413-426
TVQARXLSG AA#538-546
EESQN...QELL AA#648-662
LGFLGA...MGAAS AA#518-532
LGIWGCSGKL AA#591-600
EESQ...KDLL AA#645-660
UN
3sec
1min
30min
20hr
TD
m/z
spectrum
centroid
Binomial
distriubtion width
Bimodal(1)
Bimodal(2)

**Figure S4. Bimodal spectra suggestive of conformational switching across strains, related to Figure 6.**

Two peptides in BG505.664, five peptides in AMC008.664, and three peptides in CE1176.664 exhibited unusually broad isotopic distributions that were binomially fit to two populations (Figures A-C respectively). Each panel displays the spectra from each respective time point. The lighter in mass/more protected population is shaded in gray, and the heavier in mass/faster exchanging population is shaded in purple. The legend describing the centroid, peak envelope, and binomial fit are displayed in the bottom center of the Figure.

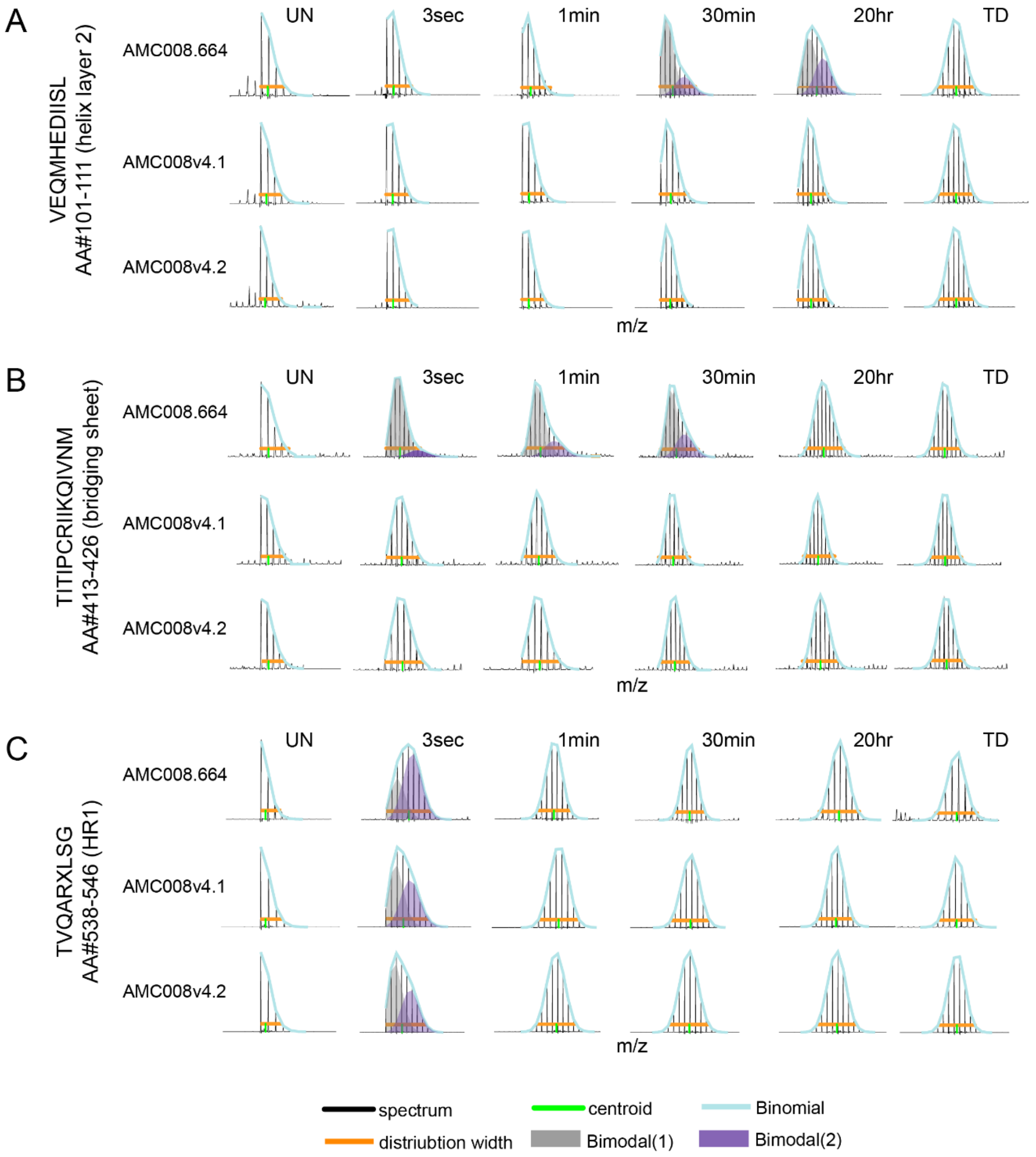


**Figure S5. Additional stabilizing mutations quench conformational sampling observed in the dynamic AMC008.664 trimer, related to Figure 7.** Mass spectra at each HDX time point of the AMC008 SOSIP constructs (.664, v4.1, and v4.2) are aligned. Spectra with a mass envelope width that was unusually broad were binomially fit to two populations. The lighter in mass/more protected population is shaded in gray, and the heavier in mass/more exposed population is shaded in purple. Peptides are highlighted spanning the gp120 inner domain helix 2 **(A)**, the bridging sheet **(B),** and HR1 in gp41 **(C).** Two of the three peptides displayed bimodal spectra only in the minimally engineered SOSIP.664 trimers, however the peptide 538-546 in gp41, where an X is used for the L543N mutation site in the v4 constructs, displayed bimodal spectra at the earliest time point in all three constructs.

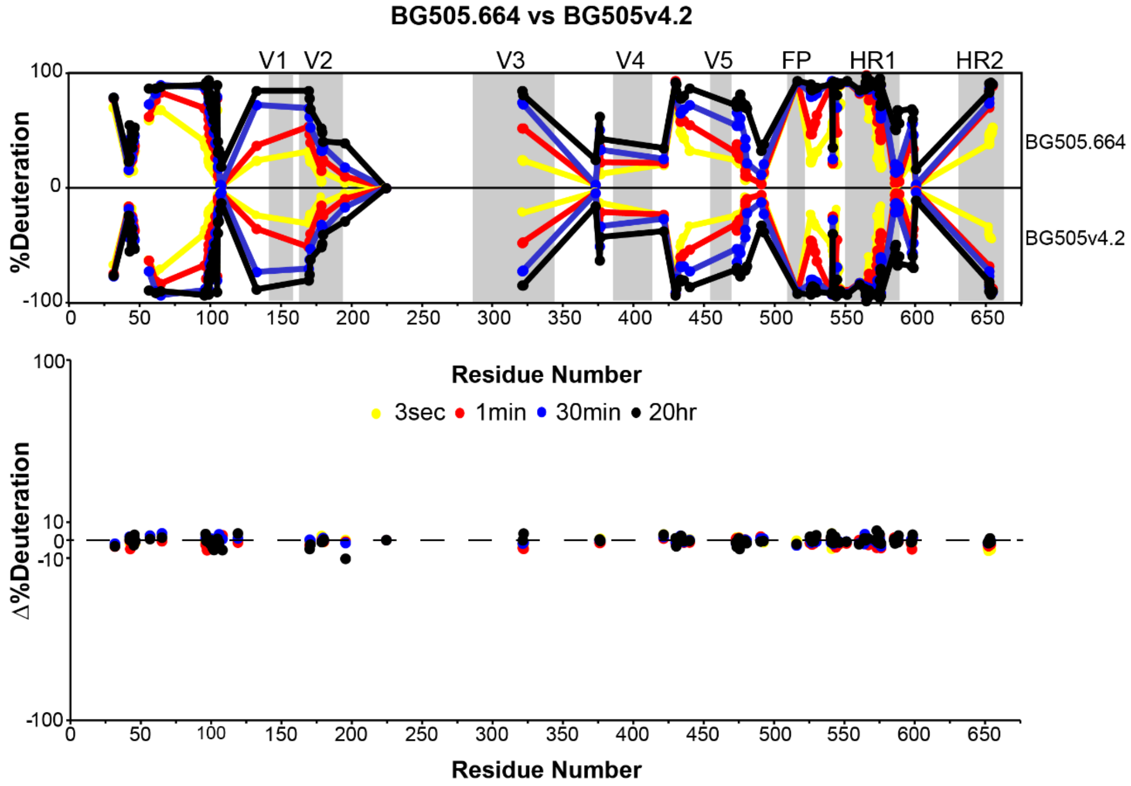


**Figure S6 BG505.664 and BG505v4.2 butterfly and differential plots, related to Figure 7 and Data S1**

The top Figure is a butterfly plot. The mid-point of each homologous peptide found in BG505.664 (top half) and BG505v4.2 (bottom half) is plotted to show deuterium uptake with time (3 second time point in yellow, 1 minute time point in red, 30 minute time point in blue, and 20 hour time point in black). The bottom Figure is a differential plot where the %difference in exchange between BG505.664 and v4.2 is plotted. Peptides falling below the 0 dashed line are regions more protected from exchange in the v4.2 construct, and peptides falling above the dashed line are regions that became less protected from exchange in the v4.2 construct. Deuterium uptake plots comparing homologous BG505.664 (blue) and BG505v4.2 (red) peptides are in an attached excel sheet (Data S1). Each data point is the average % deuteration from duplicate measurements at each time point (3sec, 1min, 30min, and 20hours) with standard deviation bars. Peptides spanning the H66R or A316W mutation site are displayed as X.

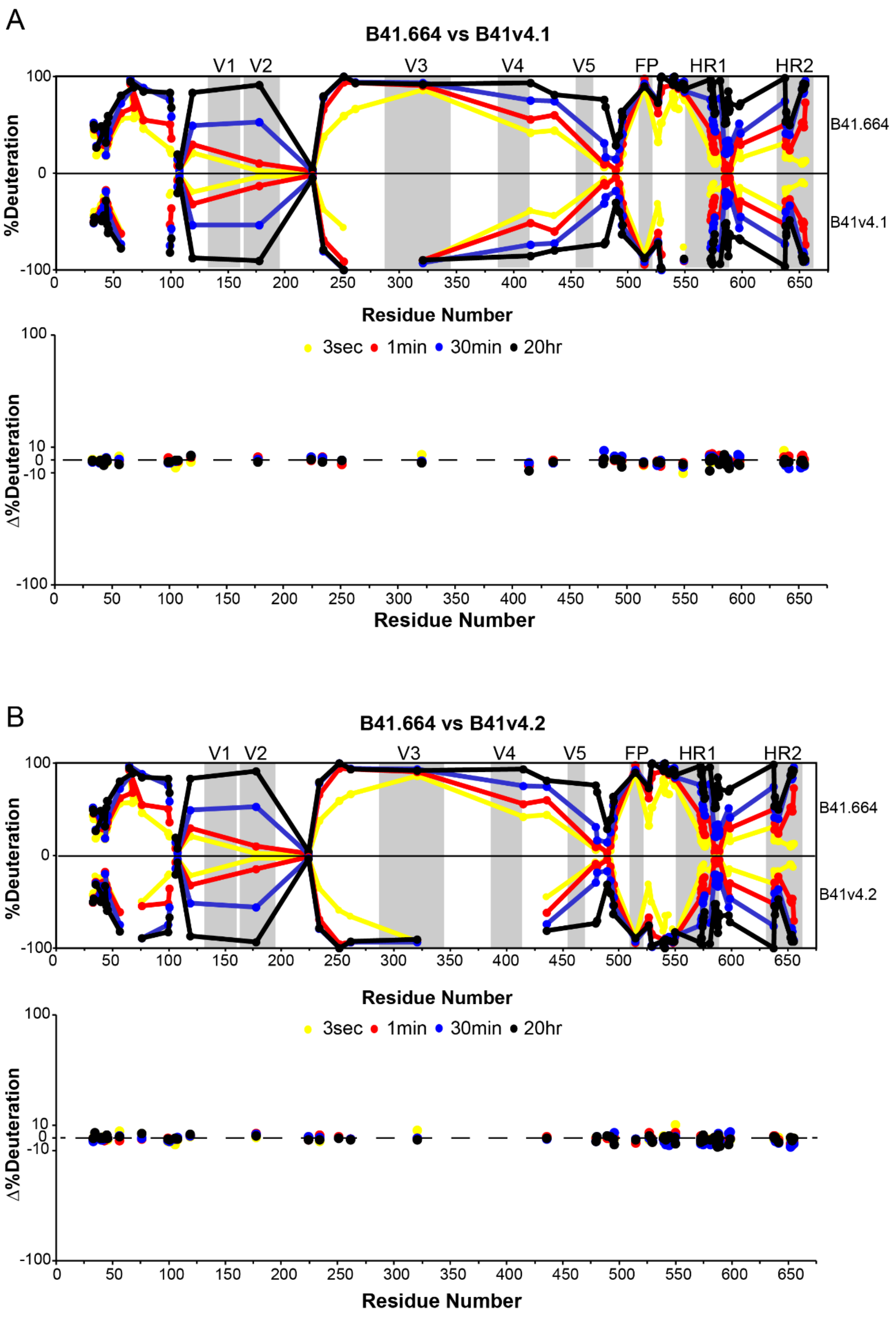
A
B41.664 vs B41v4.1
V1 V2 V3 V4 V5 FP HR1 HR2
B41.664
B41v4.1
%Deuteration
Residue Number
3sec 1min 30min 20hr
Δ%Deuteration
Residue Number
B
B41.664 vs B41v4.2
V1 V2 V3 V4 V5 FP HR1 HR2
B41.664
B41v4.2
%Deuteration
Residue Number
3sec 1min 30min 20hr
Δ%Deuteration
Residue Number

**Figure S7 B41.664, B41v4.1, and B41v4.2 butterfly plots, and differential plots, related to Figure 7 and Data S1**

(A) The top Figure is a butterfly plot. The mid-point of each homologous peptide found in B41.664 (top half) and B41v4.1 (bottom half) is plotted to show deuterium uptake with time (3 second time point in yellow, 1 minute time point in red, 30 minute time point in blue, and 20 hour time point in black). The bottom Figure is a differential plot where the %difference in exchange between B41.664 and v4.1 is plotted. Peptides falling below the 0 dashed line are regions more protected from exchange in the v4.1 construct, and peptides falling above the dashed line are regions that became less protected from exchange in the v4.1 construct. Figure (B) is similar to Figure (A), but compares B41.664 to B41v4.2. An attached excel sheet (Data S1) displays the deuterium uptake plots comparing homologous B41.664 (blue), B41v4.1 (green), and B41v4.2 (red) peptides. Each data point is the average % deuteration from duplicate measurements at each time point (3 sec, 1 min, 30 min, and 20 hours) with standard deviation bars. Peptides spanning the E64K, H66R, A316W, I535M, and L543N mutation sites are displayed as X.

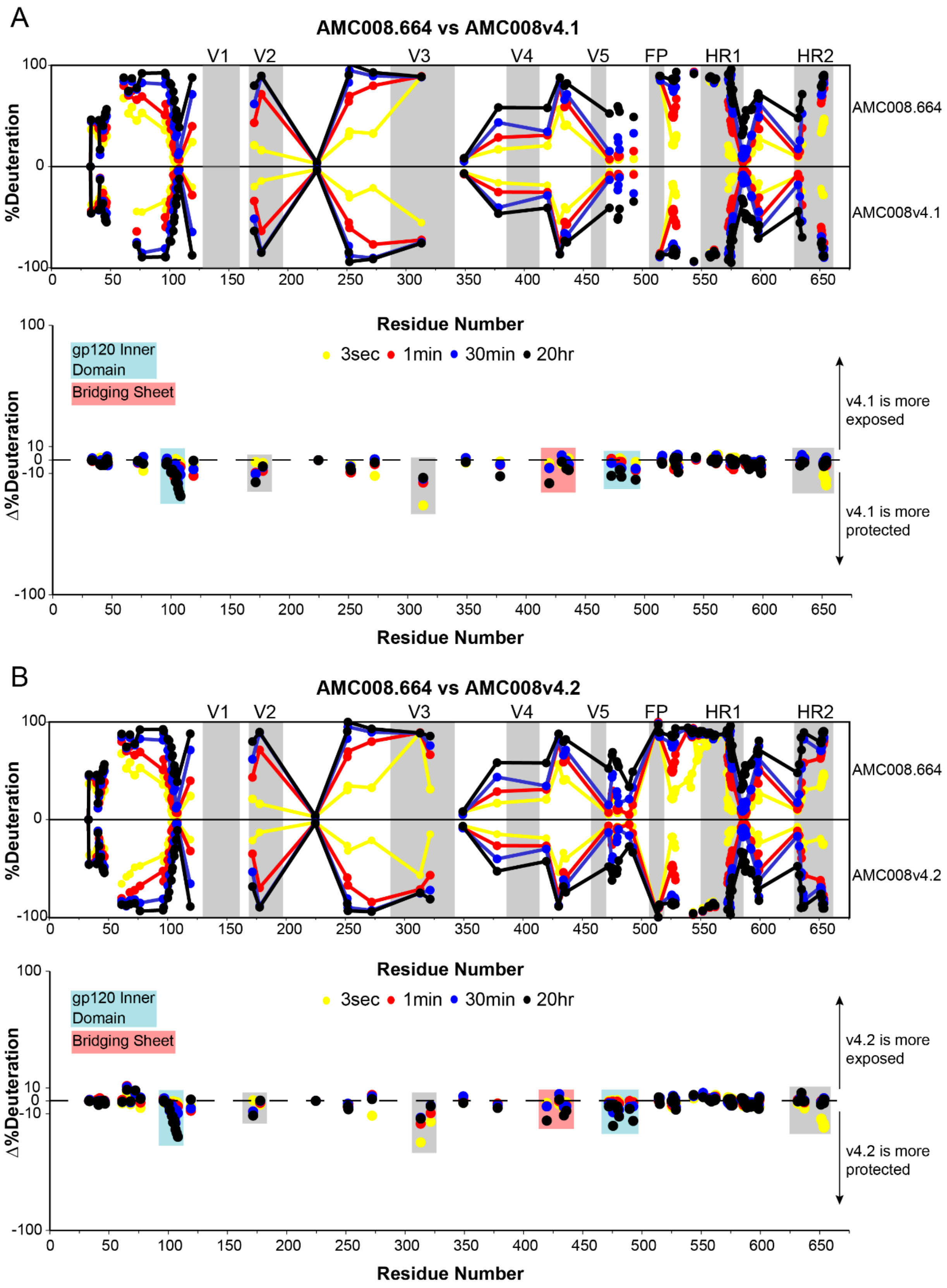

A
AMC008.664 vs AMC008v4.1
V1
V2
V3
V4
V5
FP
HR1
HR2
%Deuteration
AMC008.664
AMC008v4.1
Residue Number
3sec 1min 30min 20hr
gp120 Inner Domain
Bridging Sheet
Δ%Deuteration
v4.1 is more exposed
v4.1 is more protected
Residue Number
B
AMC008.664 vs AMC008v4.2
V1
V2
V3
V4
V5
FP
HR1
HR2
%Deuteration
AMC008.664
AMC008v4.2
Residue Number
3sec 1min 30min 20hr
gp120 Inner Domain
Bridging Sheet
Δ%Deuteration
v4.2 is more exposed
v4.2 is more protected
Residue Number

**Figure S8 AMC008.664, AMC008v4.1, and AMC008v4.2 butterfly plots, differential plots, and deuterium uptake plots, related to Figure 7 and Data S1**

(A) The top Figure is a butterfly plot. The mid-point of each homologous peptide found in AMC008.664 (top half) and AMC008v4.1 (bottom half) is plotted to show deuterium uptake with time (3 second time point in yellow, 1 minute time point in red, 30 minute time point in blue, and 20 hour time point in black). The bottom Figure is a differential plot where the %difference in exchange between AMC008.664 and v4.1 is plotted. Peptides falling below the 0 dashed line are regions more protected from exchange in the v4.1 construct, and peptides falling above the dashed line are regions that became less protected from exchange in the v4.1 construct. Figure (B) is similar to Figure (A), but compares ACM008.664 to AMC008v4.2. An attached spreadsheet (Data S1) displays the deuterium uptake plots comparing homologous AMC008.664 (blue), AMC008v4.1 (green), and AMC008v4.2 (red) peptides. Each data point is the average % deuteration from duplicate measurements at each time point (3 sec, 1 min, 30 min, and 20 hours) with standard deviation bars. Peptides spanning the E64K, H66R, A316W, I535M, and L543N mutation sites are displayed as X.

## Supplementary Tables.

| Dataset | BG505.664 | BG505v4.2 | |
|---|---|---|---|
| HDX reaction details | 85% D2O buffer, pH* 7.457, labeled at RT (23.4°C), Quenched at pH 2.51 in 200mM TCEP, 8M urea, 0.2%FA | 85% D2O buffer, pH* 7.457, labeled at RT (23.4°C), Quenched at pH 2.51 in 200mM TCEP, 8M urea, 0.2%FA | |
| HDX time course | 3 sec, 1 min, 30 min, 20 hrs | 3 sec, 1 min, 30 min, 20 hrs | |
| HDX controls | PPPI, PPPF | PPPI, PPPF | |
| Back-exchange | 10.2 +/- 7.4% | 11.5 +/- 11.4% | |
| Number of peptides | 154 | 154 | |
| Sequence coverage | 58.70% | 58.70% | |
| Average peptide length/redundancy | 12.1 residue length/ 2.8 redundancy | 12.1 residue length/2.8 redundancy | |
| Replicates (biological or technical) | two technical replicates, 3 biological replicates | two technical replicates | |
| Repeatability | stddev of 1.1% across technical replicates | stddev of 1.5% across technical replicates | |
| **Dataset** | **AMC008.664** | **AMC008v4.1** | **AMC008v4.2** |
| HDX reaction details | 85% D2O buffer, pH* 7.457, labeled at RT (23.4°C), Quenched at pH 2.51 in 200mM TCEP, 8M urea, 0.2%FA | 85% D2O buffer, pH* 7.457, labeled at RT (23.4°C), Quenched at pH 2.51 in 200mM TCEP, 8M urea, 0.2%FA | 85% D2O buffer, pH* 7.457, labeled at RT (23.4°C), Quenched at pH 2.51 in 200mM TCEP, 8M urea, 0.2%FA |
| HDX time course | 3 sec, 1 min, 30 min, 20 hrs | 3 sec, 1 min, 30 min, 20 hrs | 3 sec, 1 min, 30 min, 20 hrs |
| HDX controls | BG505.664, PPPI, PPPF | BG505.664, PPPI, PPPF | BG505.664, PPPI, PPPF |
| Back-exchange | 12.8 +/- 8.0% | 12.1 +/- 7.9% | 12.3 +/- 7.3% |
| Number of peptides | 135 | 135 | 135 |
| Sequence coverage | 61.10% | 61.10% | 61.10% |
| Average peptide length/redundancy | 13.0 residues/2.6 redundancy | 13.0 residues/2.6 redundancy | 13.0 residues/2.6 redundancy |
| Replicates (biological or technical) | two technical replicates | two technical replicates | two technical replicates |
| Repeatability | stddev of 0.90% across technical replicates | stddev of 1.1% across technical replicates | stddev of 0.96% across technical replicates |
| **Dataset** | **B41.664** | **B41v4.1** | **B41v4.2** |
| HDX reaction details | 85% D2O buffer, pH* 7.457, labeled at RT (23.4°C), Quenched at pH 2.51 in 200mM TCEP, 8M urea, 0.2%FA | 85% D2O buffer, pH* 7.457, labeled at RT (23.4°C), Quenched at pH 2.51 in 200mM TCEP, 8M urea, 0.2%FA | 85% D2O buffer, pH* 7.457, labeled at RT (23.4°C), Quenched at pH 2.51 in 200mM TCEP, 8M urea, 0.2%FA |
| HDX time course | 3 sec, 1 min, 30 min, 20 hrs | 3 sec, 1 min, 30 min, 20 hrs | 3 sec, 1 min, 30 min, 20 hrs |
| HDX controls | BG505.664, PPPI, PPPF | BG505.664, PPPI, PPPF | BG505.664, PPPI, PPPF |

| | | | |
|---|---|---|---|
| Back-exchange | 11.5 +/- 13.1% | 13.2 +/- 13.4% | 10.5 +/- 6.8% |
| Number of peptides | 89 | 89 | 89 |
| Sequence coverage | 60.60% | 60.60% | 60.60% |
| Average peptide length/redundancy | 12.0 residues/1.7 redundancy | 12.0 residues/1.7 redundancy | 12.0 residues/1.7 redundancy |
| Replicates (biological or technical) | two technical replicates | two technical replicates | two technical replicates |
| Repeatability | stddev of 1.8% across technical replicates | stddev of 1.7% across technical replicates | stddev of 0.93% across two technical replicates |
| **Dataset** | **JRFL.664** | **CE1176.664** | |
| HDX reaction details | 85% D2O buffer, pH* 7.514, labeled at RT (22.1°C), Quenched at pH 2.509 in 200mM TCEP, 8M urea, 0.2%FA | 85% D2O buffer, pH* 7.514, labeled at RT (23.3°C), Quenched at pH 2.511 in 200mM TCEP, 8M urea, 0.2%FA | |
| HDX time course | 3 sec, 1 min, 30 min, 20 hrs | 3 sec, 1 min, 30 min, 20 hrs | |
| HDX controls | BG505.664, PPPI, PPPF | BG505.664, PPPI, PPPF | |
| Back-exchange | 8.4 +/- 7.9% | 8.2 +/- 7.3% | |
| Number of peptides | 98 | 80 | |
| Sequence coverage | 60.40% | 61.00% | |
| Average peptide length/redundancy | 13.0 residues/2.0 redundancy | 13.7 residues/1.3 redundancy | |
| Replicates (biological or technical) | two technical replicates | three technical replicates | |
| Repeatability | stddev of 1.6% | stddev of 0.87% | |

**Table S1, related to Figure 2 and Data S1.**

HDX reaction details and coverage information across each SOSIP construct.

| | | | Open/Closed Conformation specific | | | | CD4 bs | V3 N332 | V3 tip specific | | | V2i | | gp120-41 |
|---|---|---|---|---|---|---|---|---|---|---|---|---|---|---|
| Tier | Clade | SOSIP | PGT145 $K_D$ | PG16 $K_D$ | b12 $K_D$ | 17b $K_D$ | VRC01 $K_D$ | PGT121 $K_D$ | 447-52D $K_D$ | 3074 $K_D$ | 3869 $K_D$ | 830A $K_D$ | 2158 $K_D$ | 35022 $K_D$ |
| 1B | B | AMC008.664 | 735.4+/- 246.9 | ND | 20.2 +/- 2.1 | 135.2 +/- 4.0 | 24.5 +/- 7.9 | 8.7 +/- 3.9 | 27.8 +/- 3.3 | 48.9 +/- 3.7 | 13.5 +/- 0.6 | ND | ND | 72.6 +/- 19.3 |
| 1B | B | AMC008v4.2 | 265.5 +/- 152.0 | ND | ND | ND | 6.5 +/- 3.9 | 2.4 +/- 1.4 | ND | ND | ND | ND | ND | 41.5 +/- 8.7 |
| 2 | B | B41.664 | 23.4 +/- 0.9 | 419 +/- 110.3 | 102.5 +/- 19.0 | ND | 90.3 +/- 10.3 | 115.5 +/- 2.1 | 125.5 +/- 21.9 | 662.5 +/- 95.5 | 97.1 +/- 22.6 | 145.0 +/- 31.1 | ND | 160.5 +/- 16.3 |
| 2 | B | B41v4.2 | 15.4 +/- 7.9 | 346.5 +/- 10.6 | 151.0 +/- 36.8 | ND | 108.9 +/- 38.4 | 91.5 +/- 38.9 | 184.2 +/- 139.8 | ND | 220.5 +/- 105.4 | 276.5 +/- 232.6 | ND | 119.0 +/- 19.7 |
| 2 | A | BG505.664 | 15.6 +/- 4.6 | 86.8 +/- 8.5 | 1114.4 +/- 315.7 | ND | 8.1 +/- 1.7 | 15.8 +/- 5.4 | 33.0 +/- 12.4 | 62.8 +/- 1.6 | 257.5 +/- 191.7 | ND | ND | 78.1 +/- 28.1 |
| 2 | A | BG505v4.2 | 13.2 +/- 5.6 | 67.4 +/- 21.0 | 120.8 +/- 20.2 | ND | 11.2 +/- 6.5 | 15.3 +/- 4.7 | ND | 6542 +/- 5412 | 1036.0 +/- 444.2 | ND | ND | 28.0 +/- 3.5 |

| | $K_D$ nM |
|---|---|
| | 0-10 |
| | 11-50 |
| | 50-250 |
| | 250-500 |
| | 500-1000 |

**Table S2: Isolate specific differences in binding affinities of Abs specific for different regions on Env, related to Figure 8**. The BLI determined average binding affinities from at least two independent experiments of each of the five isolates for a panel of Abs specific different conformations or localized regions of interest. Affinities and binding rates are colored as indicated above to highlight differences in binding affinity ($K_D$).

| | | | Open/Closed Conformation specific | | | | CD4 bs | V3 N332 | V3 tip specific | | | V2i | | gp120-41 |
|---|---|---|---|---|---|---|---|---|---|---|---|---|---|---|
| Tier | Clade | SOSIP | PGT145 $k_{on}$ (1/Ms) | PGT16 $k_{on}$ (1/Ms) | b12 $k_{on}$ (1/Ms) | 17b $k_{on}$ (1/Ms) | VRC01 $k_{on}$ (1/Ms) | PGT121 $k_{on}$ (1/Ms) | 447-52D $k_{on}$ (1/Ms) | 3074 $k_{on}$ (1/Ms) | 3869 $k_{on}$ (1/Ms) | 830A $k_{on}$ (1/Ms) | 2158 $k_{on}$ (1/Ms) | 35022 $k_{on}$ (1/Ms) |
| 1B | B | AMC008 .664 | 2.29E+03 | ND | 2.70E+04 | 4.02E+03 | 1.18E+04 | 3.92E+04 | 4.19E+04 | 3.77E+04 | 3.63E+04 | 2.58E+04 | ND | 6.52E+03 |
| 1B | B | AMC008 v4.2 | 6.30E+03 | ND | ND | ND | 1.20E+04 | 4.96E+04 | ND | ND | ND | ND | ND | 7.61E+03 |
| 2 | B | B41.664 | 2.46E+04 | 8.78E+03 | 1.08E+04 | ND | 3.98E+03 | 4.82E+03 | 6.83E+03 | 4.35E+03 | 5.59E+03 | 5.71E+03 | ND | 6.91E+03 |
| 2 | B | B41v4.2 | 3.11E+04 | 1.67E+04 | 4.70E+03 | ND | 3.81E+03 | 5.53E+03 | 6.15E+03 | ND | 3.74E+03 | 3.64E+03 | ND | 6.69E+03 |
| 2 | A | BG505.6 64 | 1.29E+05 | 3.17E+04 | 1.97E+03 | ND | 1.42E+04 | 2.09E+04 | 2.11E+03 | 3.36E+03 | 2.02E+03 | ND | ND | 6.02E+03 |
| 2 | A | BG505v 4.2 | 1.38E+05 | 5.15E+04 | 4.70E+03 | ND | 1.72E+04 | 2.15E+04 | ND | 2.25E+02 | 4.43E+02 | ND | ND | 8.33E+03 |

| | $k_{on}$ 1/Ms |
|---|---|
| | >1.0E+05 |
| | 1.0E+04 - 1.0E+05 |
| | 1.0E+03 - 1.0E+04 |
| | 1.0E+02 - 1.0E+03 |
| | <1.0E+02 |

**Table S3: Isolate specific differences in association rates of Abs specific for different regions on Env, related to Figure 8**. The BLI determined average association rates from at least two independent experiments of each of the five isolates for a panel of Abs specific different conformations or localized

regions of interest. Binding rates are colored as indicated above to highlight differences in binding association rates ($k_{on}$) across strains.

| | | | Open/Closed Conformation specific | | | | CD4 bs | V3 N332 | V3 tip specific | | | V2i | | gp120-41 |
|---|---|---|---|---|---|---|---|---|---|---|---|---|---|---|
| Tier | Clade | SOSIP | PGT145 $k_{off}$ (1/s) | PGT16 $k_{off}$ (1/s) | b12 $k_{off}$ (1/s) | 17b $k_{off}$ (1/s) | VRC01 $k_{off}$ (1/s) | PGT121 $k_{off}$ (1/s) | 447-52D $k_{off}$ (1/s) | 3074 $k_{off}$ (1/s) | 3869 $k_{off}$ (1/s) | 830A $k_{off}$ (1/s) | 2158 $k_{off}$ (1/s) | 35022 $k_{off}$ (1/s) |
| 1B | B | AMC008.664 | 1.63E-03 | ND | 5.47E-04 | 5.43E-04 | 2.89E-04 | 3.43E-04 | 1.18E-03 | 1.85E-03 | 4.86E-04 | no off rate | ND | 4.80E-04 |
| 1B | B | AMC008v4.2 | 1.44E-03 | ND | ND | ND | 7.36E-05 | 1.21E-04 | ND | ND | ND | ND | ND | 3.18E-04 |
| 2 | B | B41.664 | 5.60E-04 | 3.62E-03 | 1.11E-03 | ND | 3.54E-04 | 5.57E-04 | 8.57E-04 | 2.89E-03 | 5.35E-04 | 8.27E-04 | ND | 1.10E-03 |
| 2 | B | B41v4.2 | 5.57E-04 | 2.71E-03 | 1.08E-03 | ND | 5.92E-04 | 8.09E-05 | 1.17E-03 | ND | 9.05E-04 | ND | ND | 8.01E-04 |
| 2 | A | BG505.664 | 1.85E-03 | 2.72E-03 | 2.17E-03 | ND | 1.14E-04 | 3.53E-04 | 6.35E-05 | 2.10E-04 | 2.98E-04 | ND | ND | 4.52E-04 |
| 2 | A | BG505v4.2 | 1.68E-03 | 3.37E-03 | 5.67E-04 | ND | 1.95E-04 | 3.14E-04 | ND | 6.10E-04 | 3.81E-04 | ND | ND | 2.30E-04 |

| | $k_{off}$ (1/s) |
|---|---|
| | >1.0E-03 |
| | 1.0E-04 - 1.0E-03 |
| | <1.0E-04 |

**Table S4: Isolate specific differences in dissociation rates of Abs specific for different regions on Env, related to Figure 8**. The BLI determined average dissociation rates from at least two independent experiments of each of the five isolates for a panel of Abs specific different conformations or localized regions of interest. Dissociation rates are colored as indicated above to highlight differences dissociation rates ($k_{off}$) across strains.
